# CRISPR-Cas9-Based Knockout of the Prion Protein and Its Effect on the Proteome

**DOI:** 10.1371/journal.pone.0114594

**Published:** 2014-12-09

**Authors:** Mohadeseh Mehrabian, Dylan Brethour, Sarah MacIsaac, Jin Kyu Kim, C . Geeth Gunawardana, Hansen Wang, Gerold Schmitt-Ulms

**Affiliations:** 1 Tanz Centre for Research in Neurodegenerative Diseases, University of Toronto, Toronto, Ontario, Canada; 2 Department of Laboratory Medicine and Pathobiology, University of Toronto, Toronto, Ontario, Canada; University of Maryland School of Medicine, United States of America

## Abstract

The molecular function of the cellular prion protein (PrP^C^) and the mechanism by which it may contribute to neurotoxicity in prion diseases and Alzheimer's disease are only partially understood. Mouse neuroblastoma Neuro2a cells and, more recently, C2C12 myocytes and myotubes have emerged as popular models for investigating the cellular biology of PrP. Mouse epithelial NMuMG cells might become attractive models for studying the possible involvement of PrP in a morphogenetic program underlying epithelial-to-mesenchymal transitions. Here we describe the generation of PrP knockout clones from these cell lines using CRISPR-Cas9 knockout technology. More specifically, knockout clones were generated with two separate guide RNAs targeting recognition sites on opposite strands within the first hundred nucleotides of the *Prnp* coding sequence. Several PrP knockout clones were isolated and genomic insertions and deletions near the CRISPR-target sites were characterized. Subsequently, deep quantitative global proteome analyses that recorded the relative abundance of>3000 proteins (data deposited to ProteomeXchange Consortium) were undertaken to begin to characterize the molecular consequences of PrP deficiency. The levels of ∼120 proteins were shown to reproducibly correlate with the presence or absence of PrP, with most of these proteins belonging to extracellular components, cell junctions or the cytoskeleton.

## Introduction

The prion protein (PrP), with more than 10,000 articles published on it, several dozen ortholog PrP structures deposited in protein data bank repositories, and countless reagents in circulation to facilitate its detection and characterization, may rank amongst the most studied proteins to date. PrP gained its notoriety by the central role it was shown to play in a group of invariably fatal prion diseases that can afflict humans and some mammalian species [1]. In the disease, the cellular form of the prion protein (PrP^C^), which is widely expressed in vertebrate cells, is known to undergo a conformational change and to acquire different physicochemical properties [2]. Despite considerable interest in PrP^C^, and no shortage of possible roles that have been proposed for the protein, its physiological function has remained unclear [3]. More recently, PrP^C^ has been proposed to also play a role in Alzheimer's disease by serving as a receptor of oligomeric forms of the amyloid beta (Aβ) peptide [4], the primary constituent of amyloid plaques observed in individuals afflicted with this disease. The degree to which PrP^C^ contributes in the aforementioned neurodegenerative diseases to complex cellular etiologies that lead to neurotoxicity and, eventually, cell death has not been resolved [5].

To begin to address these and related questions, several *Prnp* knockout mouse models have been generated and closely scrutinized for phenotypes [6]. At this time, more than a dozen relatively subtle phenotypes have been reported in these PrP-deficient mice [3], yet it has been difficult to connect observations because the study of the molecular underpinnings of these phenotypes is hampered by the relative complexity of the experimental paradigms in which they were observed.

One way of reducing complexity would be to investigate cell-specific phenotypes in different cell models. The most often used and arguably best understood cell model for studying the cellular biology of PrP is the mouse neuroblastoma cell line Neuro-2a (N2a) [7], [8]. Recently, mouse C2C12 cells, a cell line of myoblasts origins, were reported to provide an attractive experimental paradigm for studying the cellular biology of PrP [9]. In light of previous reports that document a role for PrP in morphogenetic rearrangements underlying epithelial-to-mesenchymal transition (EMT) during zebrafish development [10], [11], it would further be of interest to explore the possible involvement of PrP in signaling pathways known to play a role in EMT in a mouse epithelial cell line. Mouse mammary gland-derived NMuMG cells exhibit epithelial morphology when cultured in standard growth medium but convert to a mesenchymal phenotype upon prolonged exposure to transforming growth factor beta (TGFβ) and have become a prime experimental paradigm for EMT-related studies. No PrP knockout models are available for any of the aforementioned cell models.

Until a few years ago, genomic manipulations in mammalian cells posed a formidable challenge. In recent years, genome editing methods such as those dependent on zinc-finger nucleases (ZFNs) or transcription activator-like effector nucleases (TALENs) enabled the site-specific generation of double-strand breaks. Once generated, powerful cell-encoded repair programs are initiated that lead to the non-homologous end joining (NHEJ) of breaks or to their homology-directed repair (HDR) in the presence of a template [12]. Despite the formidable advances ZFN and TALEN technologies afforded, both methods proved somewhat cumbersome and require considerable investment in time. A new technology was needed that addresses these shortcomings.

Throughout their evolution several types of bacteria have acquired the ability to fight off repeated attacks by the same virus by using an adaptive immunity. Whenever such a bacterium is invaded by a virus, it deposits short genome segments of the attacking virus in a designated location of its own genome. Should a consecutive attack by the same virus occur, the bacterium can retrieve these virus-derived fragments not only to recognize the invader but also to direct a molecular machine that can disarm the virus by cutting its genome [13], [14], [15]. Following more than 10 years of work, during which the essential molecular components of clustered regularly interspaced short palindromic repeat (CRISPR)-Cas9 machinery were defined, numerous reports published in the past year documented how this adaptive immunity can be harnessed for genome editing in a wide range of cells and organisms [16], [17], [18], [19], [20], [21], [22].

The current report describes the adaptation of this new CRISPR-Cas9 gene editing technology to ablate PrP expression in N2a neuroblastoma cells, C2C12 myoblasts and NMuMG epithelial cells. To begin to understand how PrP ablation changes the cellular proteome, a quantitative proteome investigation of a PrP knockout NMuMG clone was undertaken, with cellular extracts from PrP knockdown and wild-type NMuMG clones serving as positive and negative controls, respectively. Our analysis revealed reproducibly altered the abundance levels of ∼120 proteins in cells that exhibit no or reduced levels of PrP. A gene ontology analysis of these proteins strongly indicates a role of PrP in cellular adhesion and differentiation and identifies a majority of these proteins as belonging to the extracellular region, cell junction or the cytoskeleton.

## Results

### Strategy of CRISPR-Cas9-based PrP knockout in three mouse cell lines

**Figure 1:**
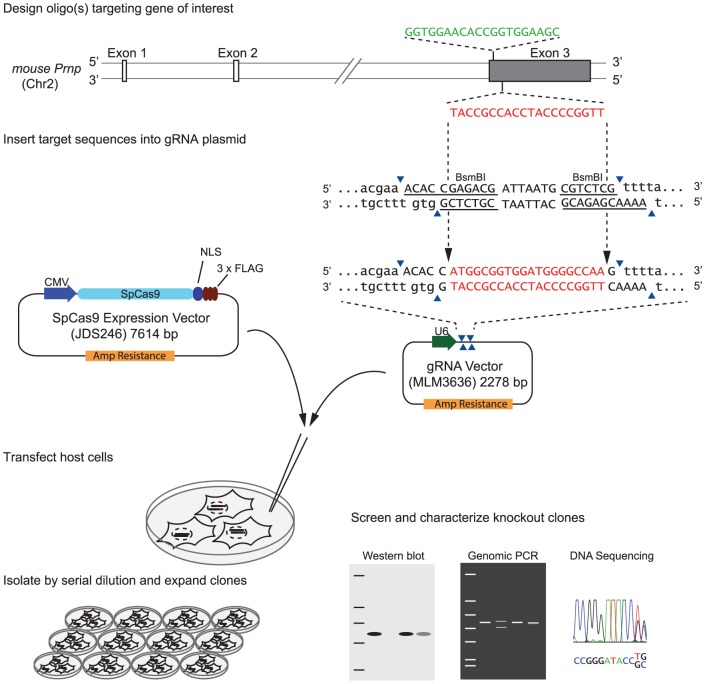
Strategy for generation of mouse PrP knockout clones based on CRISPR/Cas9-system.

Any CRISPR-Cas9-based gene knockout experiment requires that consideration be given to the choice of cell type, the reagents and strategy employed, the method for identifying positive clones and the possibility of off-target effects. The selection of N2a cells, C2C12 and NMuMG cells for this work was not only guided by the diverse systems biology these cell lines represent as neuron-, muscle- and epithelial-like cell models but also by their shared murine origins (enabling the use of identical reagents and facilitating comparative analyses across cell types) and relative ease of transfection. Given the complex karyotype of N2a cells, which comprise between 94 and 98 chromosomes in the stemline, and with anywhere between 59 and 193 chromosomes observed in individual subclones (American Type Culture Collection, Manassas, Virginia), the generation of a PrP knockout in this cell line requires the concomitant genomic editing of several *Prnp* copies. As of Spring 2013, the time at which this project was initiated, no report documenting successful genome editing of this many alleles of a single gene was available. Therefore, special consideration was given to the need to express all components of the CRISPR-Cas9 system robustly. To this end, the two-plasmid CRISPR-Cas9 system, consisting of plasmids for S*treptococcus pyogenes* Cas9 (*Sp*Cas9) and guide RNA (gRNA) expression, was adapted from the Keith Joung laboratory (Massachusetts General Hospital, MA, USA). This system drives the expression of a mammalian codon-optimized *Sp*Cas9 enzyme that was engineered to carry a mammalian nuclear localization sequence from a cytomegalovirus promoter. To further maximize the chance to produce the desired insertions or deletions (indels) at CRISPR target sites, the genome editing step was intended to stimulate NHEJ, which is known to proceed at a faster rate and is less cell cycle-dependent than genome editing approaches relying on the HDR pathway [23]. For the design of gRNAs [24], consideration was given to ensure CRISPR target sites map to an N-terminal stretch of the *Prnp* coding sequence within Exon 3. Selected sites were predicted by the ‘CRISPR Design Tool’ (http://crispr.mit.edu/) hosted by the Feng Zhang laboratory (Massachusetts Institute of Technology, MA, USA)[25] to possess a minimum number of off-target sites in the mouse genome, with particular emphasis placed on avoidance of cross-reactivity toward other coding sequences. Two sites were identified which fulfilled these criteria and also targeted opposite strands of the coding sequence (see **S1 Table** for predicted off-target sites). Following gRNA vector assembly using standard cloning procedures, host cells were co-transfected with plasmids coding for *Sp*Cas9 and one of the two gRNAs derived from the two target sites. In the absence of a suitable reporter or selection marker, the isolation of positive clones had to rely on single cell isolations by the serial (limiting) dilution followed by both PrP-specific Western blot analysis and DNA sequencing of genomic PCR products encompassing the CRISPR target sites (**Fig. 1**).

### Validation and characterization of PrP knockout cell clones

**Figure 2:**
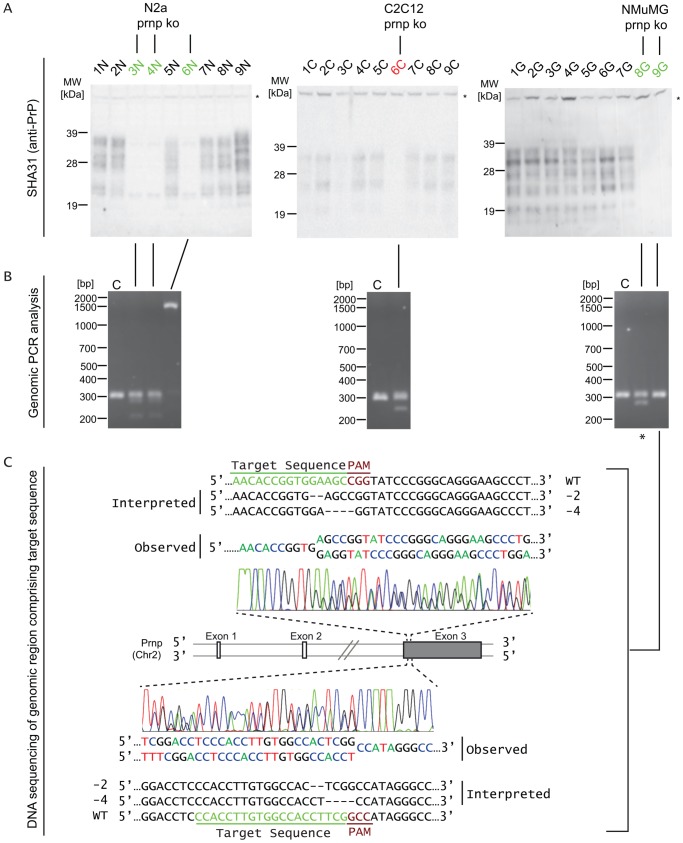
Generation of *Prnp* knockout clones in three different mouse cell lines. (**A**) Identification of clones that exhibit loss of PrP expression by Western blot analyses of cellular lysates. (**B**) Crude characterization of *Prnp* gene editing by genomic PCR analyses. Note the appearance of additional slower or faster migrating PCR products in clones that exhibit loss of PrP expression. C, control lane derived from genomic PCR analyses of cells not subjected to CRISPR/Cas9 gene editing. The asterisk identifies the NMuMG clone that was employed for the global proteome comparison described below. (**C**) Detailed insertion/deletion (indel) analysis of one *Prnp*-deficient NMuMG cell clone by DNA-sequencing of genomic PCR products. Consistent with the observed loss of detectable PrP by Western blot analysis, the analysis established the deletion of 2 and 4 nucleotides within the two *Prnp* alleles present in this NMuMG cell clone.

Whenever genomic double-strand breaks within exons are repaired by NHEJ, indels can be generated that may shift the translational reading frame by 1, 2 or 3 nucleotides. In contrast to single or double nucleotide frame shifts, the latter scenario most often merely gives rise to a loss or gain of amino acids within the natural gene product, and only rarely produces translational termination due to the insertion of a premature stop codon. Consequently, if no other limitations would apply, no more than approximately 50% of the progeny derived from a susceptible diploid parent clone could be expected to exhibit a complete loss of PrP expression. Perhaps not surprisingly, the observed yield of PrP knockout progeny was considerably lower, amounting to no more than ∼2% for C2C12 cells (1 clone amongst 44 clones tested) and ∼5% for NMuMG cells (2 clones amongst 40 clones tested). No complete knockout of PrP was achieved in N2a cells following a first round of transfection with the CRISPR-Cas9 plasmids and screening of 36 clonal isolates. A second round of transfection, however, led to ∼8.5% of N2a cell isolates (5 clones within 59 colonies tested) that exhibited no PrP expression by Western blot analysis (**Fig. 2A**).

Consistent with the intended formation of indels, genomic PCR analyses of a segment of *Prnp* Exon 3 comprising the CRISPR target sites revealed that a majority of PrP knockout clones gave rise to products that were larger or smaller than the corresponding product seen in the respective wild-type cell clones (**Fig. 2B**). Whenever such a change in the apparent molecular weight could not readily be observed, DNA sequencing revealed the PrP knockout to be reliant on small indels that are not expected to change the migration of the PCR product by agarose gel electrophoresis (**Fig. 2C**). Taken together, this phase of the project validated the generation of 8 PrP knockout clones in the three mouse cell lines. Individual clones differed from each other with respect to the CRISPR site that was targeted and the precise nature of their indels.

### Workflow of global proteome comparison of PrP knockout (or knockdown) and wild-type NMuMG cells

To begin to explore how the loss of PrP expression may alter cellular protein homeostasis, a global proteome analyses was conducted with one of the aforementioned CRISPR-Cas9-generated NMuMG cell clones exhibiting a 47-nucleotide deletion and a 5-nucleotide deletion (i.e., a 6-nucleotide deletion and a 1-nucleotide insertion) within its two *Prnp* Exon 3 alleles (**Fig. 2B**). These deletions are expected to give rise to truncated 62 and 76 amino acid *Prnp* gene products due to the formation of premature translation stop codons generated by the two frame shifts (**S1 Figure**). To generate biological starting material for this experiment, cells were grown in standard growth medium, a condition under which they are known to acquire an epithelial morphology, and were rapidly lysed in the presence of pre-heated sodium dodecyl sulfate (SDS). Three biological replicates were generated and as negative controls served naïve NMuMG cells. To avoid possible confounders caused by run-to-run variance if samples and controls were to be analyzed separately, trypsin digests of equal amounts of acetone-precipitated protein were chemically labeled with isobaric tandem mass tags (TMT). This step ensured that all biological replicates and controls could be combined and analyzed concomitantly. For the generation of technical replicates, analyses were repeated three times for each of the samples. Eluates from the separation column were directly transferred by dynamic nanospray ionization to an Orbitrap Fusion Tribrid mass spectrometer which operated in a data-dependent fragmentation mode that employed collision-induced dissociation (CID) to generate fragment ion spectra for the identification of peptides and higher-energy CID (HCD) for the detection and relative quantitation of TMT reporter ions.

**Figure 3:**
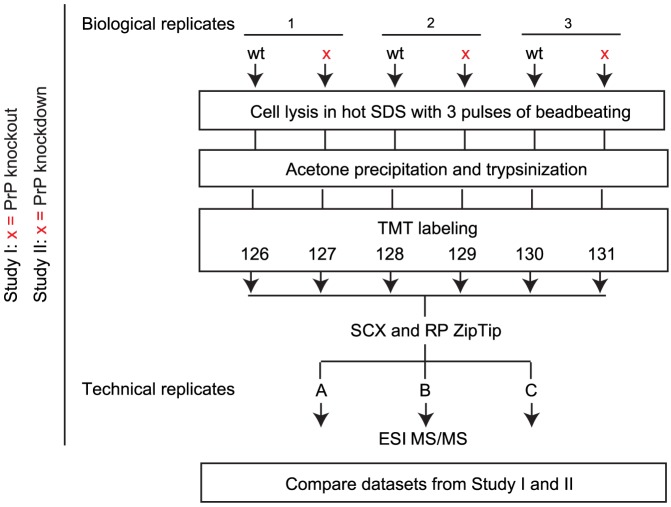
Flow-chart depicting experimental strategy for comparative analyses of the global proteomes of *Prnp* knockout (or knockdown) and wild-type NMuMG epithelial cell clones.

A caveat of the strategy outlined above was that the secretion of N-terminal PrP fragments derived from the expression of the CRISPR-edited *Prnp* gene with premature stop codons might contribute to differences between the global proteome of PrP knockout and wild-type NMuMG clones. Although there was a possibility that the cell autonomous nonsense-mediated decay program [26] could recognize the respective mutated PrP mRNAs as faulty and target them for destruction, this scenario seemed unlikely. It was dismissed largely because there is no exon-intron boundary in the *Prnp* gene in 3′ proximity to the newly inserted nonsense codons, a frequently observed requirement for triggering the nonsense-mediated decay quality control program [27]. To nevertheless determine the extent to which changes to the global proteome observed may have been caused by the loss of PrP (as opposed to be dependent on the abnormal secretion of truncated PrP fragments or idiosyncrasies of the clone or knockout method) a suitable control was needed. Note that the aforementioned caveat of N-terminally truncated PrP constructs would also exist in other CRISPR-Cas9-derived PrP knockout clones produced by employing the NHEJ pathway. To circumvent this confounder, an orthogonal set of three biological samples was instead generated from an NMuMG cell clone, which had been derived from the NMuMG parent cell line following its stable transfection with a plasmid coding for a PrP-specific shRNA. This clone exhibited more than 75% reduction in PrP expression levels when assessed by PrP-specific Western blot analysis, with relative band intensities measured on a quantitative digital image scanner. As for the CRISPR-Cas9 PrP ko clone, the total proteome of the stable PrP knockdown NMuMG cell clone was again compared to the total proteome of naïve NMuMG cells using an identical workflow to the one described above (**Fig. 3**).

### The global proteome of PrP-deficient NMuMG cells

**Figure 4:**
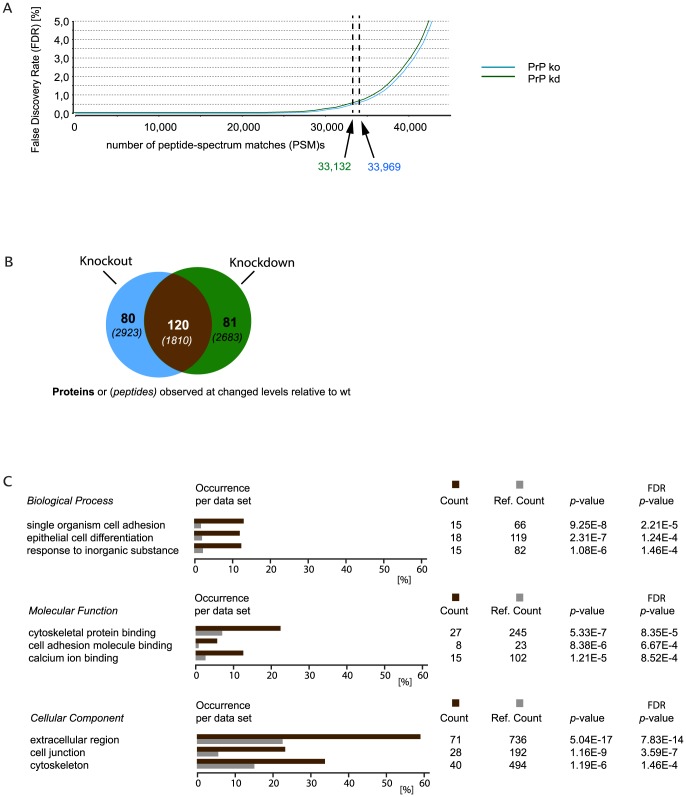
PrP deficiency generated by CRISPR/Cas9-mediated gene knockout or stable shRNA-mediated knockdown manifests in highly reproducible changes to the expression of more than hundred proteins in NMuMG cell model. (A) Chart depicting number of peptide-spectrum matches versus false discovery rate (FDR). 33,132 and 33,969 peptide-to-spectrum matches (PSMs) passed the set FDR threshold of 0.5 in PrP knockout and knockdown datasets, respectively. (B) Venn diagram depicting overlap in proteins (peptides) whose expression was altered in clones made deficient for PrP expression by the two aforementioned methods. (C) Several gene ontology (GO) classifiers were significantly overrepresented when their occurrence was compared amongst the 120 proteins whose abundance levels were consistently altered by PrP knockout (or knockdown). The list of all 3254 mouse NMuMG protein groups detected in this study served as the reference data set for these analyses. Colour code: dark brown, proteins whose abundance levels were changed in both PrP kd and ko data sets; grey, subset of proteins within reference data set assigned to a given ‘GO’ classifier.

The global proteome analyses of NMuMG cell extracts generated upward of 50,000 spectra for each of the three technical replicates. To discriminate false positive hits from correct peptide spectrum matches (PSMs) the percolator algorithm was used [28] and a false discovery rate (FDR) target of 0.5% was applied, i.e., a peptide was only considered identified when it met this stringent filter criterion. Indicative of consistent depth of coverage and data quality in PrP knockout and knockdown datasets, a similar number of ∼33,000 PSMs passed this filter amongst all acquired spectra in both datasets (**Fig. 4A**). For any protein to be considered for TMT-based quantitation, more than three unique PSMs had to support its identification and provide reporter ion profiles. Because a relatively wide isolation window of 2 m/z was applied during the selection of parent ions and the tryptic proteome digests were of high complexity, a subset of spectra were observed to be contaminated with fragments which resulted from co-isolated peaks. Therefore, an additional filter was applied, which prevented PSMs from being considered for quantitation when the intensity of an inadvertently co-isolated parent ion exceeded 30% of the intensity of the selected parent ion. For a protein to be listed, the ratio of sample/control reporter ion intensities further had to be consistently up or down in all three biological replicates. The application of these criteria resulted in a total of 3254 protein groups that were confidently identified and relatively quantified across all biological and technical replicates. Note that the term ‘protein group’ refers to the fact that, frequently, peptides detected did not uniquely identify a specific protein but were shared amongst several protein entries in the IPI database, which caused them to be recorded as a single protein group. This scenario did not only arise from protein isoforms but was occasionally also observed with gene products of paralogs or with database entries that share highly conserved modular domains, suggesting that the true number of proteins quantified must have exceeded the number of protein groups. Because the emphasis of this work was not on identifying the largest number of proteins, no attempt was made to further distinguish proteins within proteins groups, which hereafter are simply referred to as ‘proteins’. Amongst all proteins identified in the PrP knockout dataset, 201 were consistently upregulated (>1.1 ratio) or downregulated (<0.9 ratio) on the basis of the median ratio or their intensity levels (PrP knockout levels/control levels). Similarly, the stable knockdown of PrP caused 200 proteins to be shortlisted in this manner. Importantly, a majority of 120 protein groups were changed in both of the two datasets, arguing that this subset represents the most confident candidates for proteins whose abundance levels in NMuMG are influenced by the presence or absence of PrP (**Fig. 4B**). Although the abundance level changes observed were modest for a majority of these proteins, and in no instance exceeded a three-fold change, solid statistics obtained from the recording of large numbers of reporter ions provided confidence that even smaller levels of enrichment were real and not an artifact of the method (**S2 Figure**). Furthermore, none of the proteins whose abundance levels were observed to be changed in the PrP knockout clone relative to wild-type cells could be matched to the list of genes that were predicted to harbor CRISPR off-target sites (**S1 Table**). Finally, amongst the 120 shortlisted proteins the expression of only three proteins (sodium/potassium-transporting ATPase beta-1, pantetheinase precursor and ferritin light chain 1) did not follow the same trend when knockout and knockdown datasets were directly compared (**Table 1**, see also **S2 Table**).

**Table 1:** Subset of proteins observed in PrP 'ko' and 'kd' NMuMG global proteomes at levels that deviated from 'wt' levels.

						ko	data			kd	data	
						Unique				Unique		
Description	IPI	AA	Chr	TM	PP	Spectra	Cov.	wt/ko	PP	Spectra	Cov.	wt/kd
keratin, type II cytoskeletal 7	IPI00406377.3	457	15	0	82.5	34	44.2	2.495	139.6	32	46.83	2.634
galectin-2	IPI00315771.1	130	15	0	118.78	10	50.77	1.745	60.06	11	55.38	2.623
keratin, type I cytoskeletal 14	IPI00227140.1	484	11	1	212.72	23	36.36	1.484	204.38	26	28.51	2.522
Vsig1 protein	IPI00923083.1	443	X	1	37.23	8	11.06	1.869	58.28	9	8.58	2.47
cysteine-rich with EGF-like domain protein 2 precursor	IPI00111286.1	350	15	0	17.12	5	7.43	2.025	18.94	6	10.86	1.939
keratin, type I cytoskeletal 19	IPI00112947.1	403	11	0	412.98	34	52.11	1.824	453.83	32	47.15	1.855
keratin, type II cytoskeletal 8	IPI00322209.5	490	15	1	366.73	62	61.63	1.585	461.49	59	62.45	1.775
retinal dehydrogenase 1	IPI00626662.3	501	19	1	38.07	10	10.98	1.686	35.29	8	9.58	1.256
annexin A13	IPI00115275.3	317	15	0	90.27	19	36.59	1.618	35.46	16	36.59	1.368
serpin B6 isoform b	IPI00121471.1	378	13	2	120.31	15	27.78	1.315	100.81	13	20.63	1.598
mesothelin precursor	IPI00121279.1	625	17	0	48.82	18	20.8	1.601	69.74	12	17.28	1.356
protein Niban	IPI00113389.5	926	1	0	41.27	20	13.82	1.557	34.52	19	12.2	1.329
protein S100-A6	IPI00121427.1	89	3	1	53.53	9	46.07	1.169	53.82	9	37.08	1.537
charged multivesicular body protein 2b	IPI00222386.3	213	16	0	23.97	13	33.8	1.384	23.83	11	29.11	1.52
cadherin-1	IPI00318626.1	884	8	1	79.03	19	15.27	1.27	110.54	19	13.69	1.514
cytochrome b-5, isoform CRA_a	IPI00918942.1	98	18	0	31.06	3	28.57	1.482	31.48	4	40.82	1.491
heat shock 70 kDa protein 1A	IPI00798482.8	641	17	0	46.05	16	17.94	1.46	33.43	14	20.28	1.145
annexin A1	IPI00230395.5	346	19	0	356.16	27	55.2	1.422	344.93	26	52.31	1.34
thymosin beta-10	IPI00117583.3	44	6,7	0	31.39	4	47.73	1.382	31.98	5	47.73	1.251
sodium/potassium-transporting ATPase subunit beta-1	IPI00121550.3	304	1	0	33.95	11	23.68	1.38	39.59	9	24.34	0.639
desmoplakin	IPI00553419.3	2883	13	0	123.97	125	25.98	1.274	93.73	116	24.8	1.358
AHNAK nucleoprotein isoform 1	IPI00553798.2	5656	19	0	2005.8	171	42.77	1.243	1687.7	175	46.32	1.363
brain acid soluble protein 1	IPI00129519.3	226	15	0	711.17	10	37.17	1.316	712.81	12	42.92	1.319
periplakin	IPI00129193.1	1755	16	0	358.59	110	39.72	1.291	471.3	105	40.97	1.32
LIM domain and actin-binding protein 1 isoform a	IPI00759925.2	753	15	0	86.72	22	21.38	1.301	88.28	23	22.31	1.136
6-phosphogluconate dehydrogenase, decarboxylating	IPI00466919.7	483	4	3	64.11	18	22.57	1.297	90.05	21	27.33	1.08
14-3-3 protein eta	IPI00227392.5	246	5	0	236.97	17	48.37	1.281	304	19	53.66	1.113
calpain small subunit 1	IPI00830335.1	241	7	2	284.02	6	17.43	1.272	184.68	7	20.75	1.222
tight junction protein ZO-2 isoform 2	IPI00323349.2	1167	19	0	276.45	64	35.22	1.259	180.84	62	32.05	1.228
protein kinase C and casein kinase substrate in neurons 2	IPI00125880.1	486	15	0	75.41	18	24.28	1.222	74.77	15	19.96	1.206
protein arginine N-methyltransferase 1	IPI00974841.1	318	7	1	69.04	6	15.09	0.851	23.77	7	16.67	0.927
mitochondrial import receptor subunit TOM70	IPI00988528.1	611	16	1	58.61	14	13.75	0.831	55.41	11	11.13	0.955
nucleolar RNA helicase 2	IPI00652987.3	851	10	0	297.69	44	36.08	0.843	201.54	45	35.49	0.941
reticulon-4 isoform A	IPI00469392.2	1162	11	2	190.49	19	12.13	0.837	90.26	18	10.33	0.854
vinculin	IPI00405227.3	1066	14	0	1058.4	88	46.15	0.853	905.81	82	47.19	0.826
peptidyl-prolyl cis-trans isomerase D	IPI00132966.3	370	3	0	105.24	16	30.27	0.821	74.68	17	29.19	0.88
E3 ubiquitin-protein ligase HUWE1	IPI00655012.2	4377	X	11	123.25	80	11.19	0.819	201.9	68	11.22	0.906
peroxiredoxin-6 isoform X1	IPI00754071.1	200	1	0	198.5	8	30	0.814	282.69	8	33.5	0.868
phosphate carrier protein, mitochondrial precursor	IPI00124771.1	357	10	4	66.93	17	28.01	0.786	33.84	17	26.89	0.914
aldose reductase	IPI00331729.3	316	6	0	88.15	22	37.03	0.789	43.37	18	38.29	0.851
pantetheinase precursor	IPI00943453.1	512	10	2	85.79	10	15.43	0.78	67.32	6	11.13	1.181
prostate apoptosis response 4 isoform P33	IPI00816941.1	289	10	0	79.26	8	23.88	0.82	91.72	8	24.22	0.773
DNA topoisomerase 2-beta	IPI00380494.1	1612	14	1	28.71	46	17.93	0.77	36.85	55	23.39	0.823
DNA topoisomerase II	IPI00122223.1	1528	11	1	164.75	72	29.25	0.761	154.56	70	28.21	0.844
galactokinase	IPI00265025.5	392	11	1	88.18	9	18.11	0.757	75.9	12	21.68	0.875
calponin-3	IPI00119111.2	330	3	0	61.37	21	30.91	0.843	135.41	18	26.97	0.634
unnamed protein product	IPI00988446.1	488	2	2	68.42	8	12.7	0.617	48.73	12	18.44	0.881
extracellular superoxide dismutase [Cu-Zn] precursor	IPI00114319.1	251	5	0	17.07	8	11.55	0.553	30.34	3	8.37	0.699
ferritin light chain 1	IPI00608020.4	183	13	0	63.45	13	50.27	0.542	65.53	8	25.14	1.523
annexin A6 isoform b	IPI00344395.1	667	11	0	101.82	39	41.83	0.762	71.77	42	46.63	0.521

Please see **S2 Table** for full list of proteins and statistical analyses.

From a cursory examination of the datasets, it is apparent that the levels of several intermediate filament proteins (e.g., cytoskeletal keratins 7, 8, 14 and 19) and constituens of cell-cell junctions (e.g., cadherin-1, periplakin, desmoplakin, plakoglobin, catenin alpha-1 and beta-1) were higher in wild-type than in PrP-deficient NMuMG cells. A more systematic analysis of this protein list on the basis of gene ontology (GO) annotations revealed ‘Biological Processes’ related to cell adhesion, epithelial cell differentiation and response to inorganic substance were significantly enriched amongst proteins whose abundance levels were altered in PrP-deficient NMuMG cells (**Fig. 4C**). GO assignments to ‘Molecular Functions’ and ‘Cellular Components’ overrepresented amongst these proteins further characterized the phenotype of PrP-deficient NMuMG cells as being compromised in its cell adhesion and cytoskeletal biology. There are other protein expression profiles that stand out in the datasets collected. For instance, protein abundance levels of calpains were higher in wild-type than in PrP-deficient cells and, perhaps as a consequence, the levels of calponins (i.e., calponin 2 and 3), a well-known substrate of calpains, followed the opposite trend. Finally, the annexin protein family was represented by several members in the list of proteins whose abundance levels were changed, yet no consistent trend was observed amongst these annexins, i.e., expression levels of annexins A13 and A1 were observed to be robustly upregulated and, in striking contrast, annexin A6 was observed at lower levels in wild-type cells than in PrP-deficient cells.

## Discussion

This may be the first report describing the successful knockout of PrP by CRISPR-Cas9 technology. Data presented established that the methodology applied can not only achieve a PrP knockout in diploid C2C12 myoblasts and NMuMG epithelial cells but also in cells known to possess a highly complex karyotype, such as the N2a neuroblastoma cell model. To begin to characterize the impact PrP deficiency has on the cellular proteome, a deep global proteome analysis was conducted that identified more than 120 proteins whose abundance levels are modulated by PrP in the NMuMG cell model. In concordance with proposed roles of PrP in cell adhesion, the proteins whose levels were most affected by its knockout or knockdown are known to contribute to the organization of extracellular matrix, cell-cell junctions and the cytoskeleton.

The study made use of a first-generation CRISPR-Cas9 system that relied on stimulating the cell autonomous NHEJ program for the repair of double strand breaks and concomitant generation of indels. During the course of this study, a number of reports were published that investigated specificity constraints of this system and established a risk to generate off-target effects [29], [30], [31], [32], [33]. Several measures were taken to address this risk: (i) CRISPR-target sites were selected by an algorithm that suggests sites with minimal risk to generate off-target effects; (ii) individual knockout clones were generated with one of two different CRISPR target sites; (iii) the global proteomic analyses were undertaken with clones generated by CRISPR-Cas9-based knockout and shRNA-based knockdown of PrP expression; and (iv) a comparison of the list of proteins whose levels deviated between CRISPR-Cas9-based PrP-deficient and wild-type NMuMG cells with the list of predicted CRISPR off-target sites revealed no match.

The current study is not the first to have generated *in vitro* models for studying how PrP deficiency affects the biology of cells. Indeed, there is no shortage of reports that use one of several alternative approaches, including the use of primary neurons [34] or cerebellar granule cells [35] harvested from PrP-deficient mice, and the comparison of cells that are naturally devoid of PrP (e.g., Cos7 and CHO cells) [36], [37] or express low levels of PrP (e.g., NIH-3T3 and LM-TK cells)[38] versus their derivatives following PrP overexpression. The use of CRISPR-Cas9-derived PrP-deficient models is not expected to replace all of the aforementioned approaches but should complement them. In particular, the PrP-deficient N2a clones should not only facilitate efforts to study the cellular biology of PrP^C^ but also be helpful for ongoing research aimed at defining the constraints underlying its conversion in prion diseases. In past studies employing this cell line, researchers frequently had no choice but to over-express heterologous PrP constructs equipped with epitope tags [39]. Often these studies were plagued by the presence of endogenous PrP, which might have manifested, at best, as a nuisance or technical challenge or, worse, prevented studies altogether by contributing to species barrier effects [40].

Previous attempts at identifying differences in the global proteome of wild-type and *Prnp* knockout cells or tissue have led to mixed results. For instance, a comparison of the relative abundances of 1131 brain proteins by two-dimensional differential gel electrophoresis revealed no significant differences [41]. It is conceivable that existing differences between wild-type and knockout cells may be masked when a multitude of cell types present in a tissue are concomitantly analyzed by such a shotgun approach. A plausible scenario that could hinder the discovery of PrP-dependent changes would, for instance, be the presence of opposite trends in the expression of a given protein in different cell types. Similarly, a change could be masked if it exists in only a small subset of cells. These limitations might be overcome if global proteome comparisons are undertaken on homogenous preparations of cells. In fact, when cellular extracts from PrP overexpressing and wild-type SH-SY5Y cells were compared, more than a dozen changes in relative protein abundances were reported following 2D-gel electrophoresis analysis and silver-staining of protein spots [42]. A similar number of differentially expressed proteins were also observed in a recent comparison of the membrane proteomes of primary cerebral granule cells derived from *Prnp* knockout or wild-type mice [43]. Perhaps surprisingly, there is no overlap in the proteins whose expression was observed to be changed in these two paradigms or in the current study. This could be attributed to cell type-specific effects of *Prnp* ablation or to differences inherent to the methodologies employed.

The global proteomic analyses in this report benefited from mass spectrometry instrumentation whose specifications exceeded experimental setups available for previous studies. It therefore is not surprising that the current analyses afforded deeper coverage of the global proteome. It might be expected that this mere increase in depth of coverage would lead to more candidates. However, the higher proteome coverage alone is most likely not the reason for the relatively large number of proteins whose abundance levels were observed to be changed in a PrP-dependent manner in this study. This conclusion is based on the observation that a global proteome analysis of PrP knockout mouse brains conducted in parallel and with identical instrumentation led to a comparable depth of proteome coverage, yet failed to reveal a similar number of differences in expression levels (not shown).

The current study avoided gel-based workflows in order to minimize the chance to introduce inadvertent variances due to complex sample handling workflows. In contrast to 2D gel-based approaches, which rely for relative quantitation on intensity comparisons of stained protein spots, mass spectrometry-based quantitation offers critical advantages for quantitation, namely, (i) the concomitant in-solution digestion of all proteins present in the cellular extract generates a powerful reference against which the abundances of individual proteins can be compared; and (ii) because each protein is identified on the basis of several PSMs, more than one quantitation data point is generated per protein. As a result, even small changes in expression levels can produce statistically significant hits when a given protein is identified and repeatedly quantified on the basis of a large number of peptides (**S3 Figure**).

## Conclusion

It is anticipated that cell clones described in this report will be useful to a research community studying the cellular biology of the prion and its involvement in neurodegenerative diseases. The high number of changes observed in the cellular proteome of PrP-deficient NMuMG cells might make this a particularly attractive paradigm for studying signaling downstream of PrP. The CRISPR-Cas9-based procedure described here can now easily be adapted for the generation of additional PrP ko clones in other cell lines. In future studies, the risk of off-target effects could be further reduced by employing a second generation Cas9-derived nickase for genome editing and stimulation of the cell autonomous HDR pathway [29], [30], [31], [44], [45]. When combined with more conventional *in vitro* cell manipulation approaches, these new tools should hopefully facilitate the identification of both the cellular function of PrP and signaling pathways critical for neurotoxicity in AD and prion diseases.

## Materials and Methods

### Generation of gRNA Expression Vectors

The SpCas9 plasmid JDS246 (Plasmid 43861) and the gRNA expression plasmid MLM3636 (Plasmid 43860) were obtained from a non-profit plasmid share repository (Addgene, Cambridge, MA, USA). Suitable CRISPR target sites within *Prnp* Exon 3 positive and negative strands were identified using the ‘CRISPR Design Tool’ (http://crispr.mit.edu/) described in the Results section. The respective oligonucleotide pairs were obtained from Life Technologies (Burlington, ON, Canada) and were customized to include overhangs compatible for ligation into MLM3636 linearized by digestion with BsmB1 (cat. no. R0580S; New England BioLabs, Ipswich, MA, USA), a cloning site located in this vector on the 3′ side of a U6 promoter element. Oligonucleotides were phosphorylated with polynucleotide kinase (cat. no. EK0031; Fermentas, Ottawa, ON, Canada), annealed and inserted into the gRNA plasmid using T4 DNA ligase (cat no. M0202S; New England Biolabs) and transformed into Turbo competent *E. coli* (cat no. C2984H; New England Biolabs).

### Cell Culture and Transfection

Mouse neuroblastoma Neuro-2a (N2a) (CCL-131) and mouse myoblast C2C12 cells (CRL-1772) were sourced from the American Tissue Culture Collection (ATCC) (Manassas, VA, USA). Mouse mammary gland NMuMG cells were, kindly, provided by Dr. Jeffrey Wrana (University of Toronto, ON, Canada) but can also be sourced from the ATCC (CRL-1636). N2a and C2C12 cell lines were cultured as recommended by the ATCC distributor and NMuMG cells were grown in Dulbecco's Modified Eagles medium (DMEM) supplemented with 10% FBS (cat no. 12484028, Life Technologies), 1% GlutaMAX (cat. no 35050061, Life Technologies), 10 µg/mL insulin (cat no. I9278-5ML, Sigma-Aldrich, Oakville, ON, Canada) and 1% antibiotic-antimycotic solution (cat no. 15240062, Life Technologies). Transfection with 3∶1 (w/w) mixtures of SpCas9 and customized gRNA plasmids were carried out with the Lipofectamine 2000 or Lipofectamine LTX (Life Technologies) according to the manufacturer's instructions and at cell culture plate confluencies of approximately 75%. Forty-eight hours after transfection, cells were harvested, diluted in cell culture medium to a level of 1 cell/mL and replated. Once individual colonies formed, these were, initially, cultured in separate wells of 24-well plates and, subsequently, further expanded in 6-well and 60 mm plates until cell numbers were sufficient for Western blot analyses.

### Generation of stable knockdown cell clones

The shRNA vectors (TRCN0000008471 and TRCN0000008472) against PrP were obtained from the Thermo Scientific TRC Lentiviral shRNA Library (Ottawa, ON, Canada) and were co-transfected into NMuMG cells using Lipofectamine LTX (Life Technologies). The selection antibiotic, puromycin (cat no. P7255-25MG, Sigma Aldrich) was added to the medium 30h after transfection to the final concentration of 1.1 µg/mL and medium changed every 1-2 days. Following the selection, clonal isolation from the pool of stable cells was performed as described above for the knockout cells. The stable cell clones were maintained and cultured with puromycin at all times.

### Genetic analysis

To sequence the genomic region targeted by CRISPR-Cas9-mediated *Prnp* editing, genomic DNA was isolated from cells using QIAamp DNA Mini Kit (cat no. 51304, Qiagen, Valencia, CA, USA). DNA samples (20 ng/reaction) were amplified by polymerase chain reaction (PCR) using primers (5′-TCTTTGTGACTATGTGGACTG-3′ and 5′-TGCCACATGCTTGAGGTTGGTT-3′) that were predicted to anneal to *Prnp* gene sequences flanking the CRISPR target sites. The PCR conditions were 94°C for 5 min, followed by 40 cycles of 94°C for 45 s, 59°C for 45 s, 72°C for 60 s, and 7 min at 72°C. The PCR products were analyzed on an ABI PRISM 3100 Genetic Analyzer and visualized by DNA Sequencing Analysis 3.7 (Applied Biosystems).

### Western blot analyses

Cells were grown to near confluency in 60 mm plates. Growth medium was removed and the cells were rinsed twice with ice-cold phosphate buffered saline (PBS) before lysis with a buffer consisting of 50 mM Tris, pH 8.0, 150 mM NaCl, 1% NP-40, and Roche Complete Protease Inhibitor Cocktail. Insoluble cell debris was removed by 5 minute centrifugation at 14,000 rpm and 4°C. The protein levels were adjusted based on a bicinchoninic acid colourimetric assay and equal amounts of protein were separated on 4–12% or 12% Novex NuPAGE Bis-Tris gels (Life Technologies), and transferred to a 0.45 micron PVDF membrane. The blot membranes were blocked in skim milk and incubated with the primary anti- PrP antibody Sha31 (1∶5000; cat. no. A03213; Bertin Pharma, Montigny-le-Bretonneux, France) overnight at 4°C. Subsequently, blot membranes were incubated with anti-mouse secondary antibody (1∶10,000; cat no. 170-6516; Bio-Rad, Mississauga, ON, Canada) and the ECL reagent (cat. no. RPN2106; GE Healthcare, Baie d'Urfe, QC, Canada). Signals were detected with either X-ray film or the Li-COR Odyssey Fc digital imaging system (NE, USA).

### Sample preparation for comparative global proteomics analysis

For the comparative analyses of global proteomes NMuMG cell clones were employed, which expressed wild-type levels of PrP, no PrP or stably reduced levels of PrP. For each of these cell clones, three 100 mm cell culture plates grown to near confluency served as starting material for generating three biological replicates. Approximately, 5×10^6^ cells were rapidly lysed with the aid of 0.5 mm glass beads and a Mini-BeadBeater-8 (Biospec Products Inc., Oklahoma, USA) in the presence of SDS-containing Lysis Buffer (2% SDS, 62.5 mM HEPES/NaOH, pH 8.0), which had been preheated to 90°C. Following three cycles of 1 minute bead beading at ‘Homogenization Level’, samples were further incubated at 90°C to deactivate residual enzymatic activities in the cellular extracts. Protein levels were adjusted by BCA colourimetric assay (Thermo Scientific, Nepean, Ontario, Canada). Subsequently, disulfide bonds were first reduced in 100 µg aliquots for 30 min at 60°C in the presence of 5 mM tris(2-carboxyethyl) phosphine (TCEP), then alkylated for 1 hr at room temperature in the presence of 10 mM 4-vinylpyiridine (4-VP). The samples were then acetone precipitated, the pellets washed with 90% acetone and redissolved in 9 M urea. To ensure that the residual urea concentration did not exceed 1.5 M, samples were diluted in Tetraethylammonium bromide buffer (TEAB) and then digested with side chain-modified porcine trypsin overnight at 37°C. The covalent modification of peptides with reagents from the 6-plex amine-reactive tandem mass tag (TMT) labeling kit followed instructions provided by the manufacturer (Thermo Fisher Scientific, Waltham, Massachusetts, USA).

### Nanospray ionization tandem mass spectrometry

To ensure excellent proteome coverage, and to remove urea, buffer components and unreacted TMT reagents, samples were ZipTip-purified on reversed-phase (C18) and strong cation exchange resins. Eluents were adjusted to 0.1% formic acid and separated on 25 cm C18 nanocapillary columns (Acclaim PepMap RSLC with 100 Å pore size, 2 µm particle size, 75 µm inner diameter) using an EASY-nLC 1100 system (Thermo Fisher Scientific). A linear four-hour gradient of 2 to 95% acetonitrile in 0.1% formic acid was used to elute peptides and transfer them by dynamic nanospray ionization to the ion transfer tube of an Orbitrap Fusion Tribrid mass spectrometer. The acquisition method was designed with a view to maximize parallelization and involved three scan types. First, a survey scan of the 400–2000 m/z range at 120,000 resolution was conducted in the orbitrap with the automated gain control target set to 2 e^5^. Next, the most intense precursor ion carrying two or more charges was isolated, subjected to collision-induced dissociation (CID) and its fragments detected in the linear ion trap located at the back end of the machine. Finally, the ten most intense fragments from this MS2 scan were synchronously sent to the ion-routing multipole for higher-energy collision-induced dissociation (HCD). The low m/z fragments obtained during MS3 fragmentation, including the TMT reporter ions, were detected at 60,000 resolution in the orbitrap with the automatic gain control target set to 1.0 e^5^ and the maximum injection time limited to 120 ms. The combined cycle time for this method was set to 3 seconds during which as many precursors as possible, selected on the basis of their intensity and in the order from most intense to lower intense, were subjected to this processing scheme. Dynamic exclusion prevented the re-analysis of any precursor mass in a 20 ppm m/z window for the duration of 300 seconds.

### Post-acquisition analyses

For the analyses of global proteome datasets, the international protein index (IPI) mouse database (Version 3.87) was interrogated by Mascot (Version 2.4; Matrix Science Ltd, London, UK) and Sequest HT search engines embedded in Proteome Discoverer (Version 1.4; Thermo Fisher Scientific) and by integrated proteomics software packages Scaffold (Version Q+; Proteome Software Inc., Portland, Oregon, USA) and PEAKS Studio (Version 6.0; Bioinformatics Solutions Inc., Waterloo, Ontario, Canada). The percolator algorithm [28] was applied to filter all CID spectra and select the subset of spectra that exceed a false discovery rate target of 0.5. The mass spectrometry proteomics data have been deposited to the ProteomeXchange Consortium [46] via the PRIDE partner repository [47] with the dataset identifier PXD001301.

## Supporting Information

S1 Figure **Consequence of CRISPR-Cas9-mediated** ***Prnp*** **genome editing in NMuMG Clone 8G that was used for global proteome comparisons in this study.**
(PDF)

S2 Figure **Representative examples of protein quantitation data underlying identifications of proteins whose levels were changed in wild-type versus PrP knockout (knockdown) NMuMG cells.** The plots exemplify proteins whose abundance levels were observed to be (**A**) higher (keratin, type II cytoskeletal 7); (**B**) lower (annexin A6 isoform B); or (**C**) unchanged (spectrin alpha chain isoform 1) in comparisons of wild-type and PrP knockout (knockdown) cells. See also **S3** **Figure** for legend.
(PDF)

S3 Figure **Representative plots depicting quantitation data that document the ability of the proteomic workflow used in this study to distinguish even minor changes in the abundance levels of proteins.** The plots depict peptide quantitation data of proteins which passed the thresholds applied for the TMT-based abundance level ratios (>1.1 or <0.9) by only a narrow margin: (**A**) Hypoxanthine-guanine phosphoribosyltransferase, reduced in PrP-deficient cells; (**B**) CD63-antigen-like; increased in PrP-deficient cells. Note the log2 scale on the plot ordinates but the non-logarithmic presentation of Median Peptide Ratios and Inter Quartile Ranges.
(PDF)

S1 Table **Top 10 predicted off-target sites of CRISPR-Cas9 gRNAs employed in this study.**
(PDF)

S2 Table **List of all proteins observed in PrP knockout (or knockdown) NMuMG global proteomes at abundance levels that deviated from wild-type levels.** Background colours in the ‘Description’ column were applied to facilitate the recognition of similar proteins by their identical color coding.
(PDF)
