# Supplementary figures and images for: CRISPR-Cas9-Based Knockout of the Prion Protein and Its Effect on the Proteome
Source: PLoS One. 2014 Dec 9;9(12):e114594. doi: 10.1371/journal.pone.0114594 (PMC4260877; doi:10.1371/journal.pone.0114594)

### Figure S1

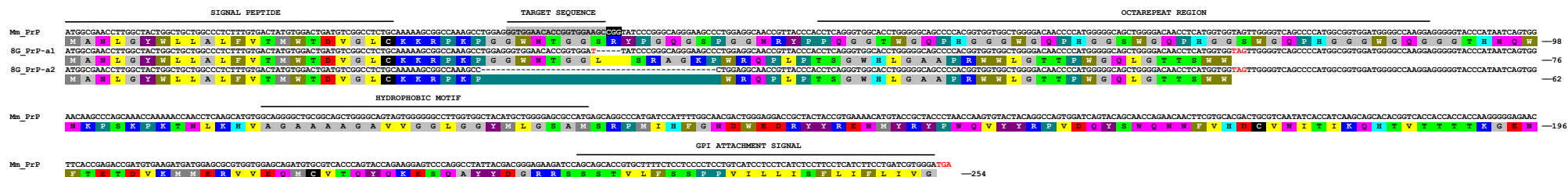

Supplement: S1 Figure — Consequence of CRISPR-Cas9-mediated Prnp genome editing in NMuMG Clone 8G that was used for global proteome comparisons in this study. (PDF) [file pone.0114594.s001.pdf]

Figure S3

**A** hypoxanthine-guanine phosphoribosyltransferase (IPI00284806.8)

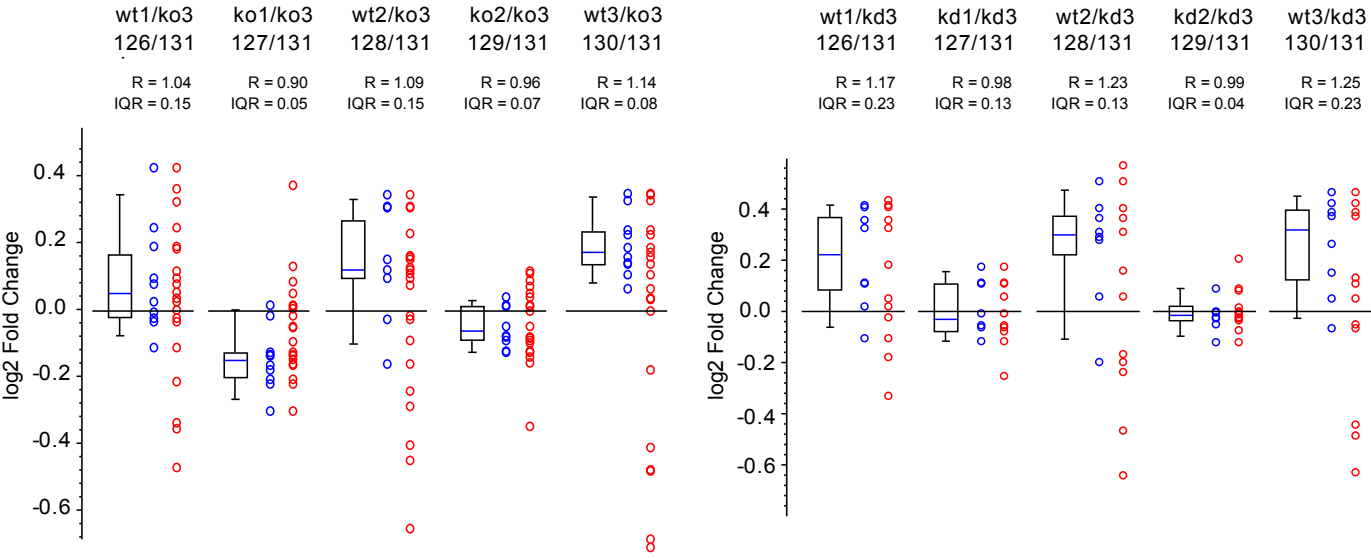

**B** CD63 antigen-like (IPI00986380.1)

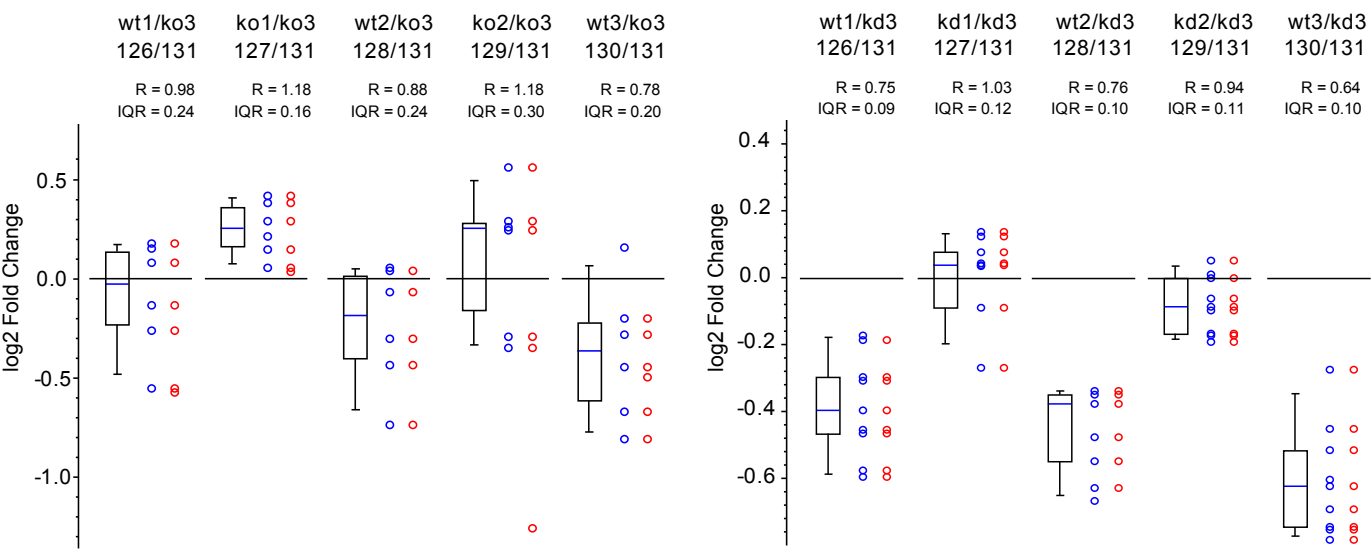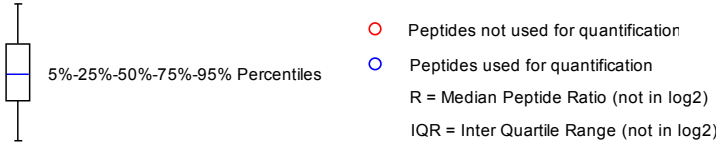

Supplement: S3 Figure — Representative plots depicting quantitation data that document the ability of the proteomic workflow used in this study to distinguish even minor changes in the abundance levels of proteins. The plots depict peptide quantitation data of proteins which passed the thresholds applied for the TMT-based abundance level ratios (>1.1 or <0.9) by only a narrow margin: (A) Hypoxanthine-guanine phosphoribosyltransferase, reduced in PrP-deficient cells; (B) CD63-antigen-like; increased in PrP-deficient cells. Note the log2 scale on the plot ordinates but the non-logarithmic presentation of Median Peptide Ratios and Inter Quartile Ranges. (PDF) [file pone.0114594.s003.pdf]
