# Supplementary material for: CRISPR-Cas9-Based Knockout of the Prion Protein and Its Effect on the Proteome
Source: PLoS One. 2014 Dec 9;9(12):e114594. doi: 10.1371/journal.pone.0114594 (PMC4260877; doi:10.1371/journal.pone.0114594)

Figure S2

keratin, type II cytoskeletal 7 (IPI00406377.3)

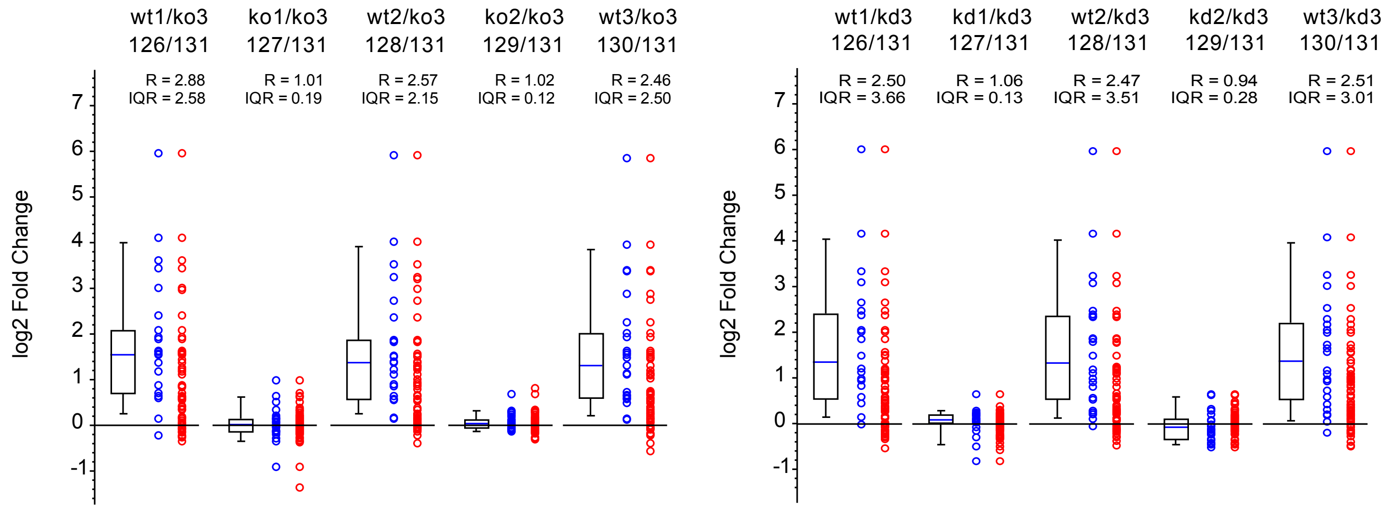

annexin A6 isoform b (IPI00310240.4)

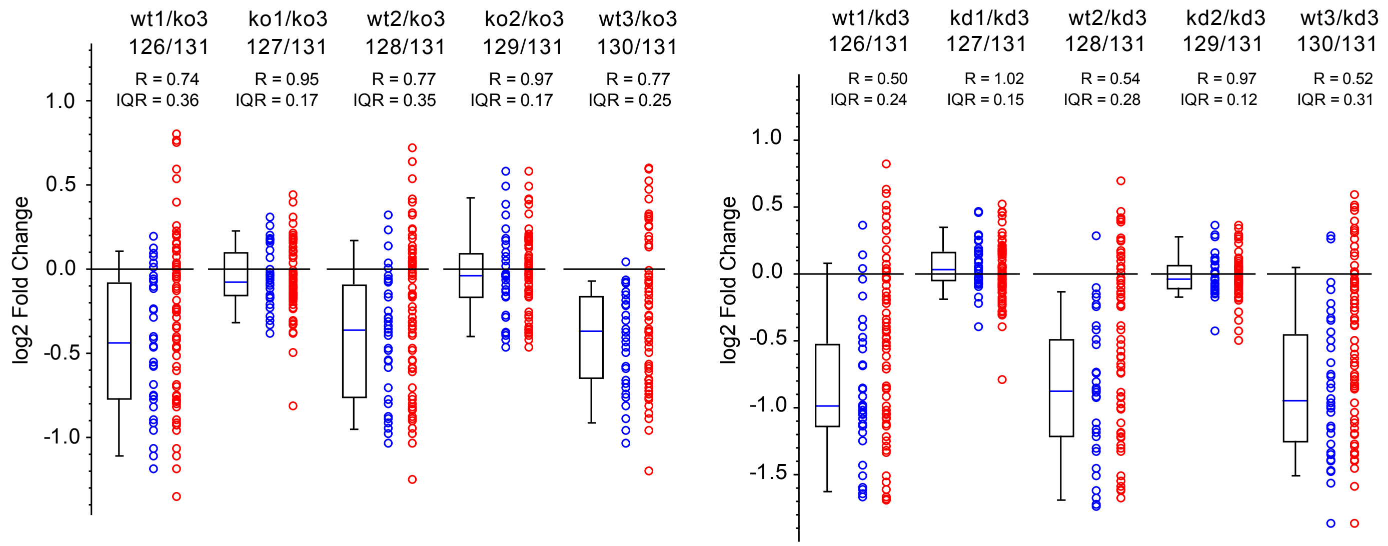

spectrin alpha chain isoform 1 (IPI00753815.3)

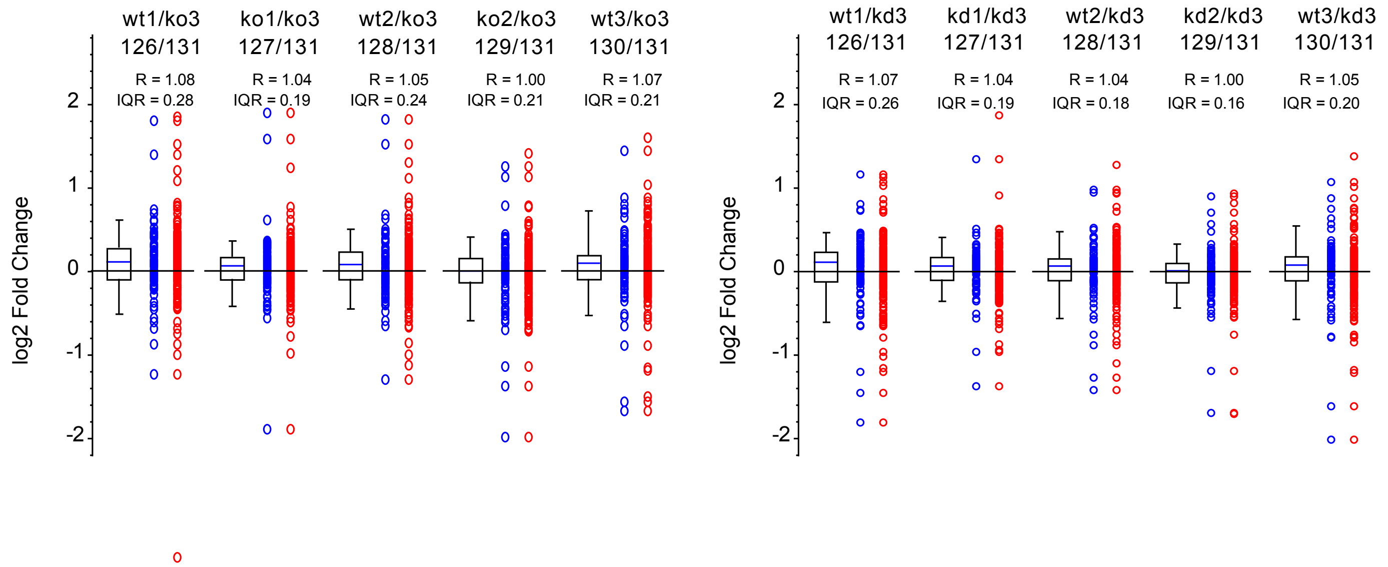

Supplement: S2 Figure — Representative examples of protein quantitation data underlying identifications of proteins whose levels were changed in wild-type versus PrP knockout (knockdown) NMuMG cells. The plots exemplify proteins whose abundance levels were observed to be (A) higher (keratin, type II cytoskeletal 7); (B) lower (annexin A6 isoform B); or (C) unchanged (spectrin alpha chain isoform 1) in comparisons of wild-type and PrP knockout (knockdown) cells. See also S3 Figure for legend. (PDF) [file pone.0114594.s002.pdf]
