# Supplementary material for: CRISPR-Cas9-Based Knockout of the Prion Protein and Its Effect on the Proteome
Source: PLoS One. 2014 Dec 9;9(12):e114594. doi: 10.1371/journal.pone.0114594 (PMC4260877; doi:10.1371/journal.pone.0114594)
Supplement: S1 Table — Top 10 predicted off-target sites of CRISPR-Cas9 gRNAs employed in this study. (PDF) [file pone.0114594.s004.pdf]

Table S1: Top 10 list of predicted off-target sites of CRISPR-Cas9 gRNAs employed in this study

Category	Sequence (NGG)	Mismatches Target / PAM	UCSC gene ID	Locus	Gene
Target	GGTGGAACACCGGTGGAAGC(CGG)	0 / Yes	NM_011170	chr2:131762254	prion gene (*Prnp*)
Predicted off-target sites	GGAGGAACACCGGTGGTACC(TGG)	3 / Yes	NM_019764	chr9:-102625906	angiomotin-like 2 (*Amotl2*)
	GCTGGAACAAGTGTGGAAGC(GAG)	4 / No	NM_027862	chr11:+115281119	ATP synthase, H+ transporting, mitochondrial F0 complex, subunit d (*Atp5h*)
	TCTGGAACACCAGTGGGAGC(TGG)	4 / Yes	NM_207682	chr4:-148562126	kinesin family member 1B (*Kif1b*)
	GGAGGAACCCAGGTGGAAGA(GAG)	4 / No	NM_008881	chr6:-89268182	plexin A1 (*Plxna1*)
	GGTGGAATGCTGGTGGGAGC(CGG)	4 / Yes	NM_001159394	chr16:+55818089	nuclear factor of kappa light polypeptide gene enhancer in B cells inhibitor, zeta (*Nfkbiz*)
	GGAGGAATGCCGGAGGAAGC(CAG)	4 / No	NM_001114339	chr19:+34953785	pantothenate kinase 1 (*Pank1*)
	GGTGGAAAGCCAGTTGAAGC(CGG)	4 / Yes	NM_001033285	chr1:-182064480	CDC42 binding protein kinase alpha (*Cdc42bpa*)
	GGTGGAGCAGCCGTGGAGGC(AGG)	4 / Yes	NM_001130030	chr4:-133768349	cation channel, sperm associated 4 (*Catsper4*)
	GCTGGAGCACTGGAGGAAGC(CAG)	4 / No	NM_173425	chr1:-80210386	family with sequence similarity 124, member B (*Fam124b*)
	GGTGGAACCCCTTTGGAAGG(GAG)	4 / No	NM_028838	chr9:+110854156	leucine rich repeat containing 2 (*Lrrc2*)
Target	TTGGCCCCATCCACCGCCAT(GGG)	0 / Yes	NM_011170	chr2:131762254	prion gene (*Prnp*)
Predicted off-target sites	ATGCCCTCATCCACCGCCAC(CAG)	4 / No	NM_145582	chr7:-50930753	cytosolic thiouridylase subunit 1 homolog (S. pombe) (*Ctu1*)
	TCCGCCCCCTCCTCCGCCAT(TGG)	4 / Yes	NR_045715	chr10:-126632643	RIKEN cDNA F420014N23 gene (*F420014N23Rik*)
	AAGGACCCATCCACCGCGAT(CAG)	4 / No	NM_001037801	chr19:-10872901	CD6 antigen transcript variant 1 (*Cd6*)
	GGGGCCCCTTCCACCCCCAT(CAG)	4 / No	NM_008535	chr8:+87225294	lymphoblastomic leukemia 1 (*Lyl1*)
	TTGGCACCTGCCACAGCCAT(TGG)	4 / Yes	NM_025683	chr1:-66761187	ribulose-5-phosphate-3-epimerase (*Rpe*)
	TTGACTCCATCCACAGCCAC(CAG)	4 / No	NM_001164533	chr3:+88732175	death associated protein 3 (*Dap3*)
	ATGCCCCCATCCACCGCAGT(GAG)	4 / No	NM_001003672	chr18:-37304293	protocadherin alpha subfamily C, 2 (*Pcdhac2*)
	TGGGCCACATCCACCTCCAC(TGG)	4 / Yes	NM_194333	chr1:+75122208	solute carrier family 23 (nucleobase transporters), member 3 (*Slc23a3*)
	TTGTCCCCAGCCACCACCGT(GGG)	4 / Yes	NM_011170	chr2:-131762336	prion protein (*Prnp*)
	TTGGTCCCATCCACAGCCCG(GAG)	4 / No	NM_026886	chr5:-116899560	serine/arginine repetitive matrix 4 (*Srrm4*)
