# Supplementary material for: CRISPR-Cas9-Based Knockout of the Prion Protein and Its Effect on the Proteome
Source: PLoS One. 2014 Dec 9;9(12):e114594. doi: 10.1371/journal.pone.0114594 (PMC4260877; doi:10.1371/journal.pone.0114594)
Supplement: S2 Table — List of all proteins observed in PrP knockout (or knockdown) NMuMG global proteomes at abundance levels that deviated from wild-type levels. Background colours in the ‘Description’ column were applied to facilitate the recognition of similar proteins by their identical color coding. (PDF) [file pone.0114594.s005.pdf]

Table S2: Proteins observed in PrP 'ko' or 'kd' NMuMG global proteomes at levels that deviated from 'wt' levels

Description	IPI	AA	Chr	ko	kd	TM	ko PP	ko Unique Spectra	ko Unique Sequ.	ko Cov.	ko TMT ratios 126/131	ko TMT ratios 127/131	ko TMT ratios 128/131	ko TMT ratios 129/131	ko TMT ratios 130/131	kd PP	kd Unique Spectra	kd Unique Sequ.	kd Cov.	kd TMT ratios 126/131	kd TMT ratios 127/131	kd TMT ratios 128/131	kd TMT ratios 129/131	kd TMT ratios 130/131	ko StdDev for quantitation 126/131	ko StdDev 127/131	ko StdDev 128/131	ko StdDev 129/131	ko StdDev 130/131	kd StdDev for quantitation 126/131	kd StdDev 127/131	kd StdDev 128/131	kd StdDev 129/131	kd StdDev 130/131	ko # of Pept.	kd # of Pept.
galectin-2	IPI00315771.1	130	15	X	X	0	118.78	10	6	50.77	1.67	0.95	1.65	0.97	1.91	60.06	11	7	55.4	2.53	0.91	2.40	0.96	2.94	72.52	8.97	71.49	11.62	114.81	166.24	11.98	100.10	21.46	215.11	22	15
keratin, type II cytoskeletal 7	IPI00406377.3	457	15	X	X	0	82.5	34	26	44.2	2.50	1.06	2.47	0.94	2.51	139.6	32	27	46.8	2.88	1.01	2.57	1.02	2.46	285.61	11.27	328.80	24.56	270.09	295.58	14.73	249.22	9.38	204.74	24	25
keratin, type I cytoskeletal 14	IPI00227140.1	484	11	X	X	1	212.72	23	22	36.36	1.46	1.04	1.57	1.01	1.42	204.4	26	19	28.5	2.74	1.02	2.34	1.04	2.49	69.03	11.02	88.30	14.37	52.80	114.32	38.48	172.60	29.61	162.74	17	11
Cysteine And Glycine-Rich Protein 2 CSRP2 = LIM Domain Only Protein 5	IPI00988582.1	155	10	X		0	29.53	6	6	38.71	2.43	0.92	2.10	0.96	2.73										62.41	10.64	96.53	21.07	102.14						4	
Vsig1 protein	IPI00923083.1	443	X	X	X	1	37.23	8	6	11.06	1.74	1.12	1.91	1.03	1.95	58.28	9	5	8.58	2.57	1.05	2.46	1.01	2.38	55.86	17.22	78.90	9.25	91.49	135.51	19.19	156.25	10.86	155.77	13	12
keratin, type I cytoskeletal 42	IPI00468696.3	452	11	X		0	84.66	19	16	26.55	2.40	0.94	2.33	0.94	2.44										246.32	14.12	156.11	9.19	138.77						7	
isoform 3 of Transcription factor 25	IPI00776033.1	611		X		1	38.48	12	11	14.08	2.36	1.01	2.20	1.07	1.87										118.44	4.64	258.82	27.68	146.40						3	
neural cell adhesion molecule 1	IPI00122971.2	1115	9	X	X	2	16.08	16	14	13.27	1.10	1.02	1.05	0.98	1.00	63.19	22	19	17.2	1.96	1.02	1.90	0.96	1.87	5.38	5.87	5.13	0.95	3.46	11.63	7.20	23.99	5.67	22.58	4	6
glutathione S-transferase A4	IPI00323911.3	222	9	X	X	0										23.28	6	6	21.2	1.94	0.95	1.87	1.01	2.19						24.49	0.76	56.44	9.01	40.85		5
cysteine-rich with EGF-like domain protein 2 precursor	IPI00111286.1	350	15	X	X	0	17.12	5	3	7.43	2.17	0.98	1.79	0.79	2.11	18.94	6	4	10.9	1.91	0.94	2.00	1.01	1.91	3.30	12.52	27.68	9.84	6.46	27.57	2.60	111.63	1.21	101.69	3	4
keratin, type II cytoskeletal 79	IPI00124499.2	531	15	X		0	48.63	19	17	27.31	2.01	1.15	2.08	1.07	1.86										44.44	16.07	40.04	14.69	37.73						15	
four and a half LIM domains protein 1 isoform 1	IPI00776276.1	323	X		X	0										47.02	8	8	21.7	1.56	0.55	2.02	0.77	1.89						95.88	24.45	57.33	24.49	83.68		4
keratin, type II cytoskeletal 1b	IPI00462140.1	572	15	X		2	57.61	31	20	30.42	1.90	1.12	2.01	1.06	1.75										60.46	18.62	70.25	12.57	51.10						18	
keratin, type I cytoskeletal 19	IPI00112947.1	403	11	X	X	0	412.98	34	26	52.11	1.86	1.02	1.79	1.02	1.82	453.8	32	24	47.2	1.85	0.98	1.84	1.02	1.87	120.31	17.32	90.57	15.05	100.75	141.48	13.22	135.89	11.75	148.44	79	72
keratin, type I cytoskeletal 15	IPI00876179.1	452	11		X	1										41.53	14	11	24.8	1.75	1.03	1.83	1.05	1.74						237.28	8.63	136.73	22.24	156.22		4
uncharacterized protein LOC239673	IPI00351880.1	538	15		X	0										40.16	20	16	21.2	1.82	1.09	1.73	1.02	1.81						79.00	5.97	53.67	13.39	59.27		7
keratin, type II cytoskeletal 8	IPI00322209.5	490	15	X	X	1	366.73	62	45	61.63	1.62	1.08	1.62	1.04	1.52	461.5	59	44	62.5	1.82	1.04	1.82	1.02	1.69	44.82	14.40	44.29	13.79	37.93	78.38	17.71	65.08	12.38	67.48	102	107
retinal dehydrogenase 1	IPI00626662.3	501	19	X	X	1	38.07	10	6	10.98	1.60	1.05	1.67	0.94	1.79	35.29	8	6	9.58	1.30	0.98	1.29	1.06	1.18	34.31	11.68	55.28	8.68	37.30	37.31	16.75	16.38	9.82	26.45	6	8
zinc finger protein 593	IPI00321357.2	134	4	X	X	0	37.52	5	4	30.6	1.14	0.95	1.23	0.96	1.17	41.17	6	5	29.9	1.45	1.02	1.75	1.01	1.68	31.06	42.52	81.69	23.61	46.41	68.46	44.76	28.49	30.27	22.57	11	7
annexin A13	IPI00115275.3	317	15	X	X	0	90.27	19	15	36.59	1.74	1.02	1.53	1.05	1.58	35.46	16	14	36.6	1.40	1.00	1.40	1.02	1.30	163.04	7.65	93.59	8.28	103.91	46.37	5.34	18.05	11.07	27.29	12	12
syndecan-1	IPI00125849.1	267	12	X	X	0	34.61	5	4	13.86	1.00	0.99	0.88	0.93	1.00	57.79	6	5	16.5	1.76	1.04	1.71	1.10	1.68	7.30	6.35	5.88	4.01	5.04	75.77	3.65	22.25	6.08	50.97	5	6
mesothelin precursor	IPI00121279.1	625	17	X	X	0	48.82	18	17	20.8	1.69	1.03	1.52	1.01	1.59	69.74	12	12	17.3	1.40	1.02	1.33	0.97	1.34	54.63	9.28	51.61	12.77	35.31	25.67	7.78	27.08	5.96	29.09	12	11
annexin A8 isoform 1	IPI00169939.1	327	14		X	0										52.78	19	16	34.9	1.42	1.01	1.51	1.06	1.69						41.20	18.44	38.23	14.88	60.74		12
epidermal growth factor receptor kinase substrate 8-like protein 2	IPI00115492.1	729	7	X	X	0	137.13	28	22	26.61	1.16	1.04	1.13	1.04	1.16	130.3	26	21	24.3	1.64	1.04	1.66	0.98	1.69	18.52	7.30	16.74	12.31	7.42	43.37	20.21	45.89	21.09	42.52	13	15
protein S100-A6	IPI00121427.1	89	3	X	X	0	53.53	9	7	46.07	1.27	0.84	0.94	0.95	1.30	53.82	9	7	37.1	1.44	0.97	1.49	1.13	1.68	32.25	26.36	34.45	29.08	27.85	41.02	8.32	23.32	30.22	75.70	15	13
aldehyde dehydrogenase, mitochondrial precursor	IPI00111218.1	519	5	X	X	0	196.1	19	16	26.59	1.04	1.03	0.98	1.03	1.01	167.9	11	9	16.4	1.67	1.04	1.40	0.95	1.49	10.48	17.39	16.40	13.83	16.38	51.81	14.94	34.03	9.55	35.80	17	13
serpin B6 isoform b	IPI00121471.1	378	13	X	X	0	120.31	15	13	27.78	1.29	0.94	1.32	0.96	1.34	100.8	13	10	20.6	1.54	1.03	1.61	0.99	1.64	17.16	5.13	20.39	9.26	21.98	23.11	5.68	19.54	6.13	36.86	25	20
dihydropyrimidinase-related protein 3 isoform 1	IPI00881485.1	568	18		X	0										95.2	17	15	21.3	1.42	0.98	1.64	1.04	1.42						47.52	22.90	66.92	11.97	67.38		14
CD2-associated protein	IPI01008700.1	637	17	X	X	0	88.5	25	20	28.73	1.19	0.94	1.11	0.99	1.07	41.81	22	17	24.5	1.64	0.99	1.48	0.98	1.60	25.40	9.58	28.63	8.23	21.76	56.18	9.23	67.86	14.39	51.83	24	14
protein Niban	IPI00113389.5	926	1	X	X	0	41.27	20	15	13.82	1.53	0.99	1.61	1.06	1.53	34.52	19	14	12.2	1.31	0.95	1.38	0.98	1.30	21.25	18.74	46.02	9.91	17.56	26.03	20.43	38.12	11.42	26.59	5	6
pro-cathepsin H preproprotein	IPI00884472.1	333	9	X	X	0	26.61	7	6	12.61	1.15	0.91	1.07	0.97	1.15	32.05	12	8	17.1	1.58	1.09	1.42	0.97	1.60	13.22	13.95	15.52	4.37	13.07	78.60	4.14	48.31	16.87	55.84	9	6
heat shock 70 kDa protein 1A	IPI00798482.8	641	17	X	X	0	46.05	16	15	17.94	1.35	0.95	1.58	1.03	1.45	33.43	14	13	20.3	1.19	0.96	1.13	1.02	1.11	40.08	12.40	67.25	10.73	62.13	21.61	14.94	25.07	13.05	16.14	14	13
calcium-binding mitochondrial carrier protein SCaMC-1	IPI00468924.5	475	3	X	X	0	34.82	18	16	28.21	1.20	1.03	1.14	1.03	1.16	33.87	19	17	29.9	1.57	1.07	1.46	1.06	1.56	11.88	5.95	15.46	4.92	20.97	11.39	9.67	22.85	10.51	26.37	11	12
cadherin-1	IPI00318626.1	884	8	X	X	0	79.03	19	18	15.27	1.25	1.09	1.27	1.04	1.29	110.5	19	17	13.7	1.50	1.04	1.47	0.98	1.57	10.15	14.60	14.73	9.29	23.69	18.66	9.77	19.97	7.32	29.39	18	11
cytochrome b-5, isoform CRA_a	IPI00918942.1	98	18	X	X	0	31.06	3	3	28.57	1.53	1.04	1.45	1.02	1.47	31.48	4	4	40.8	1.45	0.99	1.54	1.01	1.49	9.22	2.05	14.85	10.60	11.25	41.54	6.72	14.41	6.90	26.12	6	5
charged multivesicular body protein 2b	IPI00222386.3	213	16	X	X	0	23.97	13	9	33.8	1.37	0.93	1.42	0.88	1.36	23.83	11	8	29.1	1.50	1.02	1.52	1.04	1.54	1.70	5.71	9.98	10.37	8.08	10.43	7.35	13.12	15.03	16.72	5	9
phosphoprotein enriched in astrocytes 15, isoform CRA_c = PEA15	IPI00336400.1	108	1		X	0										16.05	4	3	19.4	1.19	0.98	1.54	0.98	1.51						18.34	8.77	18.34	1.44	24.55		2
peripherin	IPI00129527.3	475	15		X	0										42.99	22	22	31.2	1.53	1.02	1.41	1.03	1.40						18.36	9.85	21.05	5.88	22.73		14
major vault protein	IPI00986977.1	861	7	X	X	0	105.71	18	17	18.47	1.25	1.00	1.20	1.02	1.22	97.23	27	22	21.4	1.37	0.99	1.31	0.97	1.52	35.58	10.45	25.84	13.23	27.97	47.86	9.91	42.92	15.10	46.62	16	14
annexin A1	IPI00230395.5	346	19	X	X	0	356.16	27	25	55.2	1.35	0.96	1.41	0.95	1.50	344.9	26	26	52.3	1.30	1.02	1.28	1.01	1.44	25.72	12.28	31.40	10.73	30.82	25.12	10.06	18.46	8.62	20.23	44	42
keratin, type I cytoskeletal 18	IPI00311493.5	423	15	X	X	0	326.63	46	37	51.3	1.25	1.07	1.31	1.07	1.19	181.9	40	32	47.3	1.50	1.05	1.53	1.05	1.37	31.96	14.67	35.26	13.06	33.19	53.61	15.49	56.89	13.70	39.58	75	64
sodium/potassium-transporting ATPase subunit beta-1	IPI00121550.3	304	1	X	X	0	33.95	11	8	23.68	1.49	1.00	1.26	0.98	1.40	39.59	9	8	24.3	0.56	0.93	0.63	0.98	0.73	60.70	4.28	74.93	7.60	63.51	9.18	21.48	20.18	9.30	28.54	6	11
thymosin beta-10	IPI00117583.3	44	6,7	X	X	0	31.39	4	4	47.73	1.29	1.01	1.38	1.00	1.47	31.98	5	5	47.7	1.21	1.09	1.25	1.01	1.30	20.14	10.63	18.32	5.78	26.74	24.14	17.62	19.88	11.68	33.01	16	18
desmoplakin	IPI00553419.3	2883	13	X	X	0	123.97	125	101	25.98	1.26	0.99	1.28	1.00	1.29	93.73	116	98	24.8	1.45	1.00	1.30	1.08	1.33	30.40	14.20	28.67	17.45	43.73	66.59	12.69	46.54	19.16	55.22	29	30
junction plakoglobin	IPI00229475.1	745	11	X	X	0	145.91	32	23	29.13	1.19	1.03	1.22	0.95	1.21	166.5	32	24	30.9	1.44	1.00	1.32	1.02	1.37	33.64	24.45	33.66	15.34	29.09	32.71	10.16	47.40	12.44	26.45	34	21
protein S100-A11-like	IPI00987238.1	96	4	X	X	0	77.18	7	3	27.08	1.24	0.85	1.26	0.95	1.44	41.37	4	4	28.1	1.44	1.00	1.23	0.93	1.38	14.11	7.64	18.84	9.17	26.02						8	1
catalase	IPI00869393.1	527	2	X	X	0	56.98	15	12	16.32	1.23	0.98	1.28	1.02	1.30	49.01	16	13	20.5	1.42	0.82	1.44	0.90	1.18	31.93	12.45	26.83	4.00	22.73	33.28	5.29	10.43	5.72	55.06	6	3
unnamed protein product = Actin-related protein 2/3 complex subunit 5	IPI00652873.2	92	1		X	0										21.7	6	5	37	1.43	0.90	1.37	0.94	1.41						18.92	6.63	34.08	10.69	30.50		7
NADP-dependent malic enzyme isoform 2	IPI00987914.1	552	9		X	0										112.9	19	17	23.9	1.39	1.03	1.42	1.01	1.46						35.45	8.97	30.55	13.77	43.20		19
unconventional myosin-Ie	IPI00330649.4	1107	9		X	0										36.97	26	21	16.4	1.39	1.08	1.33	1.07	1.41						2.77	8.32	8.07	23.65	26.67		5
brain acid soluble protein 1	IPI00129519.3	226	15	X	X	0	711.17	10	8	37.17	1.38	1.00	1.33	1.01	1.24	712.8	12	11	42.9	1.41	1.01	1.27	1.02	1.28	39.20	17.09	38.86	20.21	38.12	33.26	14.25	35.01	12.84	32.72	38	38
F-actin-capping protein subunit beta isoform d	IPI00776140.1	260	4		X	0										47.79	19	16	47.3	1.36	1.00	1.39	1.05	1.33						25.98	11.56	23.88	11.17	21.98		14
PREDICTED: integrin alpha-3 isoform X1	IPI00468674.5	1068	11		X	0										26.18	18	17	14.1	1.36	1.07	1.26	1.07	1.29						25.30	5.02	17.57	6.16	14.58		9
UDP-glucose 4-epimerase	IPI00153129.1	347	4	X		0	18.57	7	7	17.58	1.35	0.85	1.41	0.95	1.44										2.14	9.73	8.23	7.36	33.14						3	
astrocytic phosphoprotein PEA-15 isoform 2	IPI00121013.1	130	1	X		0	55.32	7	4	30	1.33	0.99	1.39	1.10	1.36										27.58	9.74	13.35	41.74	21.57						5	
histone H1.0	IPI00467914.3	194	15		X	0										122.2	24	22	61.3	1.33	0.99	1.42	0.95	1.29						26.80	16.68	35.36	8.29	16.54		23
actin-related protein 2/3 complex subunit 4 isoform 1	IPI00138691.6	168	6		X	0										36.72	9	8	35.7	1.32	1.08	1.17	1.02	1.20						43.98	15.67	16.68	13.64	40.16		8
periplakin	IPI00129193.1	1755	16	X	X	0	358.59	110	84	39.72	1.32	1.04	1.31	1.03	1.25	471.3	105	87	41	1.33	1.02	1.35	1.00	1.29	40.27	12.52	28.54	18.09	39.71	35.32	15.80	26.60	11.44	29.82	69	75
LIM domain and actin-binding protein 1 isoform a	IPI00759925.2	753	15	X	X	0	86.72	22	19	21.38	1.31	1.01	1.29	0.98	1.31	88.28	23	21	22.3	1.10	1.00	1.17	0.98	1.13	40.87	14.90	26.90	16.89	37.10	24.22	13.76	21.56	13.51	26.10	20	16
V-type proton ATPase subunit E 1	IPI00409922.1	226	6		X	0										20.12	10	6	24.3	1.30	1.00	1.32	1.00	1.34						28.55	14.11	11.95	14.50	14.03		6
PREDICTED: unconventional myosin-Id isoform X1	IPI00226551.2	944	11	X		0	122.44	33	29	25.21	1.30	1.06	1.28	0.94	1.17										32.02	24.72	30.71	20.37	7.67						8	
mitochondrial fission 1 protein isoform 2	IPI00857192.1	145	5	X		0	223.54	4	3	22.76	1.29	1.03	1.14	1.06	1.27										52.68	16.65	45.31	16.46	48.19						12	
non-specific lipid-transfer protein	IPI00134131.2	547	4	X		0	19.59	14	12	18.1	1.29	1.09	1.31	1.00	1.28										24.71	8.61	12.02	15.53	15.87						11	
uncharacterized protein (Fragment) = galectin 3	IPI00989544.2	177	14		X	0										173.2	13	9	34.5	1.29	0.95	1.34	0.99	1.36						44.08	7.82	40.03	11.27	36.83		39
protein flightless-1 homolog	IPI00467104.2	1271	11		X	0										85.14	37	28	17.5	1.29	1.04	1.26	0.99	1.22						22.66	10.43	20.42	12.64	16.05		16
glutathione reductase, mitochondrial precursor	IPI00331282.1	500	8		X	0										24.49	11	8	12.4	1.29	1.00	1.17	1.03	1.28						33.03	15.87	48.25	10.90	40.46		6
calpain-1 catalytic subunit	IPI00626909.1	713	19	X		0	49.78	19	17	20.9	1.28	1.07	1.23	1.03	1.26										23.08	22.09	23.40	14.95	19.67						10	
AHNAK nucleoprotein isoform 1	IPI00553798.2	5656	19	X	X	0	2005.8	171	143	42.77	1.28	1.03	1.25	1.03	1.21	1688	175	153	46.3	1.39	1.02	1.37	0.99	1.33	24.30	11.70	21.96	13.30	20.39	29.64	11.78	30.58	10.57	26.02	290	264
serine (or cysteine) proteinase inhibitor, clade B, member 6b	IPI00116247.1	377	13		X	0										84.34	8	7	16.7	1.28	1.02	1.29	0.93	1.38						30.34	4.62	28.32	15.76	43.54		5
large neutral amino acids transporter small subunit 1	IPI00331577.5	512	8		X	0										146.9	9	7	13.3	1.28	1.03	1.09	1.04	1.16						25.38	14.34	18.26	20.88	18.26		14
PDZ and LIM domain protein 7 isoform a	IPI00130607.8	457	13	X		0	29.01	6	5	12.04	1.27	1.08	1.28	0.93	1.29										0.69	6.77	7.18	9.33	2.65						3	
tight junction protein ZO-2 isoform 2	IPI00323349.2	1167	19	X	X	0	276.45	64	54	35.22	1.27	1.01	1.30	0.99	1.21	180.8	62	49	32.1	1.25	1.01	1.23	1.00	1.21	14.27	12.74	22.04	16.35	17.08	17.63	11.19	23.37	15.94	22.69	46	43
14-3-3 protein eta	IPI00227392.5	246	5	X	X	0	236.97	17	14	48.37	1.26	0.99	1.27	1.04	1.31	304	19	15	53.7	1.10	1.00	1.09	1.00	1.15	31.91	10.97	22.23	8.12	30.97	18.50	5.85	13.03	8.44	10.29	35	30
6-phosphogluconate dehydrogenase, decarboxylating	IPI00466919.7	483	4	X	X	0	64.11	18	15	22.57	1.26	0.92	1.31	1.01	1.32	90.05	21	17	27.3	1.03	0.96	1.11	1.01	1.11	31.87	8.11	29.60	8.70	25.21	14.11	9.39	17.58	4.80	13.08	14	18
protein kinase C and casein kinase substrate in neurons protein 2	IPI00125880.1	486	15	X	X	0	75.41	18	15	24.28	1.26	0.95	1.22	0.97	1.19	74.77	15	13	20	1.17	0.94	1.24	0.90	1.21	25.02	13.18	33.21	19.39	23.30	40.54	9.27	23.66	16.52	25.75	13	12
elongation of very long chain fatty acids protein 1 isoform 2	IPI00648941.1	202	4		X	0										138.9	6	1	5.94	1.26	0.93	1.13	1.08	1.20						33.46	19.23	25.51	5.59	39.71		9
unnamed protein product = peroxiredoxin 5	IPI00475031.1	211	19		X	0										53.36	11	9	35.6	1.26	1.00	1.29	1.02	1.36						32.26	9.56	36.92	12.29	20.10		14
protein-glutamine gamma-glutamyltransferase 2	IPI00387333.4	686	2		X	0										122.8	15	15	20.7	1.26	0.94	1.40	0.99	1.38						32.74	13.88	16.99	19.32	28.72		12
calpain small subunit 1	IPI00830335.1	241	7	X	X	0	284.02	6	5	17.43	1.25	0.88	1.25	0.91	1.31	184.7	7	6	20.8	1.11	0.92	1.21	0.92	1.34	17.03	6.85	12.93	16.28	21.73	16.49	5.07	14.10	11.79	23.76	13	11
actin-related protein 2/3 complex subunit 5	IPI00399943.3	151	1	X		0	28.58	8	7	28.48	1.25	0.94	1.08	0.94	1.18										11.23	6.64	8.88	10.60	10.28						6	
histone H1.3	IPI00331597.6	221	13	X	X	0	545.86	28	24	69.68	1.25	1.11	1.14	1.06	1.13	510.8	32	27	74.7	1.10	1.05	1.07	1.01	1.02	22.72	15.28	22.52	11.98	16.01	15.01	13.74	14.45	9.40	11.71	102	93
ladinin-1	IPI00319558.1	528	1	X	X	0	271.97	47	38	47.35	1.25	0.99	1.21	1.00	1.28	300.2	49	37	43.9	1.33	1.00	1.43	1.03	1.38	29.17	11.84	27.43	14.94	23.49	30.11	12.46	51.40	15.31	37.11	26	32
cell division control protein 42 homolog isoform 1 precursor	IPI00113849.1	191	4	X	X	0	219.65	9	7	32.46	1.25	0.97	1.19	0.97	1.29	150	9	6	33.5	1.22	1.07	1.23	1.00	1.16	17.28	10.70	10.22	12.17	11.97	20.21	17.33	17.25	11.76	14.70	26	26
histone H1.2	IPI00223713.5	212	13	X	X	0	543.9	27	23	68.4	1.25	1.10	1.12	1.04	1.11	536.2	27	23	66.5	1.10	1.05	1.08	1.01	1.02	26.08	15.33	25.72	14.63	18.66	16.33	13.97	16.50	10.50	13.53	72	62
histone H1.4	IPI00223714.5	219	13	X		0	428.43	34	29	77.17	1.25	1.12	1.16	1.07	1.14										25.40	15.13	25.28	13.21	18.34						93	
leucine-rich repeat flightless-interacting protein 1 isoform 1	IPI00654388.2	729	1		X	0										46.41	14	10	11.8	1.25	0.98	1.35	1.02	1.22						21.75	6.35	20.44	8.13	23.05		9
annexin A2	IPI00468203.3	339	9		X	0										105.9	25	24	42.5	1.25	1.00	1.32	0.97	1.28						24.33	12.88	25.55	10.83	23.09		40
ubiquitin-conjugating enzyme E2 T	IPI00131962.1	204	1		X	0										14.24	6	6	24.5	1.25	1.00	1.38	0.86	1.29						27.64	3.52	18.87	29.17	40.36		5
septin-2 isoform b	IPI00930754.1	321	1		X	0										91.46	15	11	26.8	1.25	0.99	1.16	1.01	1.20						12.50	13.18	12.19	9.87	9.01		16
huntingtin-interacting protein 1-related protein	IPI00876343.1	1068	5	X	X	0	117.23	41	33	25.84	1.24	1.02	1.32	1.02	1.26	92	47	37	28.9	1.10	1.09	1.19	0.95	1.10	16.93	14.16	16.41	19.26	23.37	15.07	8.17	13.51	11.50	15.26	13	12
peroxiredoxin-1	IPI00121788.1	199	4	X	X	0	197.13	16	11	43.72	1.24	0.97	1.30	0.99	1.29	102	10	9	38.2	1.18	0.99	1.28	1.00	1.21	17.32	7.79	19.68	7.32	16.54	20.59	8.27	20.23	5.04	18.33	31	20
catenin alpha-1	IPI00112963.1	906	18	X	X	0	165.09	40	30	29.03	1.23	1.03	1.21	1.03	1.21	116.6	35	30	25.3	1.15	1.01	1.11	0.98	1.11	19.10	9.73	20.07	14.29	25.54	13.27	9.65	19.46	9.06	20.49	50	40
unconventional myosin-XVIIIa isoform 2	IPI00622556.4	2035	11		X	0										146.8	52	46	19.3	1.23	0.94	1.30	0.94	1.22						40.16	15.76	39.90	28.31	39.06		21
UDP-glucose 6-dehydrogenase	IPI00118344.1	493	5	X	X	0	139.49	22	19	33.47	1.22	0.96	1.15	0.94	1.13	97.25	19	17	32.9	1.22	0.98	1.36	0.95	1.19	22.39	9.42	23.74	15.86	22.87	34.78	15.05	40.09	9.82	31.63	23	19
cytosolic acyl coenzyme A thioester hydrolase isoform 2	IPI00672508.1	379	4	X		0	136.33	11	8	18.21	1.22	0.94	1.42	0.99	1.35										25.32	4.63	11.65	17.04	23.88						9	
calreticulin precursor	IPI00123639.1	416	8	X	X	0	180.82	16	14	25.48	1.22	1.02	1.26	1.04	1.23	145.9	17	16	24.5	1.20	1.04	1.14	1.01	1.12	23.04	9.12	14.67	10.23	22.35	11.64	10.35	9.97	8.73	12.59	41	32
transmembrane emp24 domain-containing protein 10 precursor	IPI00466570.4	219	12	X	X	0	48.55	7	5	19.63	1.22	1.04	1.11	1.03	1.08	22.96	9	6	21.9	1.01	0.96	1.05	0.97	0.99	18.25	9.56	16.98	6.08	13.50	20.91	7.78	12.54	10.94	17.92	24	15
plastin-3	IPI00115528.3	630	X		X	0										99.96	26	22	32.2	1.22	0.97	1.18	0.97	1.20						23.42	11.79	26.22	10.09	16.36		23
cofilin-1-like	IPI00848816.1	166	6	X		0	103.99	10	10	45.18	1.21	0.99	1.21	1.00	1.16										14.43	8.76	21.39	11.31	19.13						28	
galectin-9 isoform 2	IPI00230649.1	322	11	X	X	0	86.19	9	6	15.84	1.21	0.99	1.27	1.04	1.25	130.6	7	6	16.2	1.14	0.95	1.16	0.95	1.21	31.64	17.12	28.55	17.01	16.80	19.87	16.96	15.11	11.91	22.40	10	9
calpain-2 catalytic subunit	IPI00308938.6	700	1	X	X	0	237.92	9	9	12.43	1.21	0.97	1.19	0.97	1.18	194.1	8	8	10.1	1.22	0.94	1.14	1.04	1.25	18.73	15.30	14.94	8.54	14.39	36.05	26.89	26.96	12.49	42.16	21	16
cathepsin D precursor	IPI00111013.1	410	7	X		0	57.87	6	5	10.24	1.21	0.96	1.27	0.99	1.18										8.77	4.80	15.30	13.63	12.96						3	
catenin beta-1	IPI00125899.1	781	9	X	X	0	183.5	26	21	27.91	1.21	1.05	1.10	1.01	1.14	182.7	32	25	32	1.10	1.01	1.07	1.02	1.11	34.85	14.94	24.99	11.24	23.97	21.94	15.43	26.12	8.93	15.35	24	18
alpha-actinin-4	IPI00118899.1	912	7	X		0	621.18	43	38	36.73	1.21	1.05	1.14	1.03	1.18										24.68	11.90	24.48	12.52	28.59						81	
Purine nucleoside phosphorylase	IPI00315452.5	289	14		X	0										54.65	14	12	34.3	1.21	0.97	1.26	0.91	1.15						31.57	11.01	28.22	14.90	23.69		12
filamin-B isoform a	IPI01007906.1	2602	14		X	0										836.3	90	74	25.3	1.21	1.00	1.16	1.00	1.16						20.62	9.71	18.58	9.95	16.60		148
GTP:AMP phosphotransferase AK3, mitochondrial	IPI00221769.5	227	19	X		0	131.42	17	14	51.54	1.20	1.07	1.09	1.01	1.17										31.81	11.55	24.78	15.57	21.66						22	
peroxiredoxin-5, mitochondrial precursor	IPI00129517.1	210	19	X		0	101.04	11	9	40	1.20	0.94	1.28	1.01	1.27										24.88	9.07	22.67	6.68	27.80						23	
rho-related GTP-binding protein RhoG precursor	IPI00116558.1	191	7	X		0	47.69	7	6	16.75	1.20	0.92	1.17	0.93	1.29										9.69	4.01	6.02	3.77	4.42						8	
MAP7 domain-containing protein 1 isoform 1	IPI00282957.4	846	4		X	0										103.6	33	33	29.6	1.20	0.91	1.32	1.03	1.15						36.15	36.01	24.85	29.34	25.89		14
cytosolic purine 5'-nucleotidase isoform 1	IPI00944164.1	586	19		X	0										42.83	13	10	12.8	1.20	0.99	1.26	1.02	1.28						17.20	12.93	18.46	11.31	7.71		11
heterogeneous nuclear ribonucleoprotein U-like protein 2	IPI01027807.1	745	19	X		0	189.64	39	30	30.74	1.19	0.98	1.17	0.98	1.14										40.74	11.78	32.35	19.48	31.88						31	
ezrin	IPI00330862.5	586	17	X	X	0	167.5	55	49	46.76	1.19	0.96	1.20	0.98	1.22	150	53	47	46.1	1.27	1.03	1.34	1.00	1.32	24.70	13.59	35.38	14.79	30.81	30.75	13.79	30.46	13.64	30.78	78	59
protein disulfide-isomerase precursor	IPI00133522.2	509	11	X	X	0	169.76	28	24	36.54	1.19	1.06	1.21	1.02	1.15	178.4	34	29	41.1	1.12	1.05	1.16	1.00	1.11	22.51	9.05	16.51	11.52	17.38	17.47	8.57	18.55	5.90	13.55	58	55
myosin regulatory light chain 12B	IPI00132705.1	172	17	X		0	56.01	6	5	26.16	1.19	1.00	1.18	1.01	1.19										10.83	2.77	6.72	4.90	12.95						7	
V-type proton ATPase catalytic subunit A	IPI00407692.3	617	16		X	0										66.05	18	15	24.2	1.19	0.97	1.15	0.90	1.17						20.97	8.99	19.84	11.05	31.17		16
tropomodulin-3	IPI00119478.1	352	9		X	0										66.38	17	12	28.1	1.19	0.94	1.24	1.01	1.18						25.34	13.42	20.48	7.38	22.91		15
transcriptional activator protein Pur-beta	IPI00128867.3	324	11	X		0	406.98	9	8	22.84	1.18	1.04	1.19	1.02	1.13										20.03	10.52	18.63	17.26	17.53						11	
UMP-CMP kinase	IPI00331146.5	227	4	X	X	0	128.12	14	8	30.4	1.18	0.81	1.19	0.96	1.34	205.4	11	7	30.4	1.24	0.96	1.41	1.04	1.28	30.67	20.40	34.49	14.76	21.59	29.45	18.52	27.30	13.17	44.41	14	17
ras-related C3 botulinum toxin substrate 1 precursor	IPI00761613.1	192	5	X	X	0	95.59	4	4	22.4	1.18	0.98	1.18	0.99	1.28	69.87	5	5	26.6	1.17	1.00	1.15	0.99	1.21	24.05	10.30	13.43	10.28	14.88	17.19	12.46	12.73	9.35	14.26	23	19
14-3-3 protein zeta/delta isoform 1	IPI00116498.1	245	15	X		0	415.29	29	24	64.9	1.18	0.94	1.21	1.01	1.21										24.30	7.87	23.72	9.68	20.55						56	
coactosin-like protein	IPI00132575.3	142	8		X	0										90.82	11	9	44.4	1.18	0.98	1.18	0.97	1.32						15.17	9.73	32.72	11.97	23.24		10
elongation of very long chain fatty acids protein 1 isoform 1	IPI00124382.1	279	4	X		0	87.75	5	2	6.09	1.17	1.12	1.21	1.06	1.21										16.37	26.40	19.30	13.29	24.44						5	
actin-related protein 2/3 complex subunit 3	IPI00124829.5	178	5	X	X	0	31.04	14	11	38.76	1.17	0.93	1.20	0.98	1.21	22.55	14	11	49.4	1.35	1.04	1.28	1.06	1.36	42.45	15.33	31.20	12.53	35.31	34.71	7.62	35.29	13.11	56.52	7	5
isoform 2 of Rab GDP dissociation inhibitor beta	IPI00416577.1	409	13	X		0	86.51	17	14	30.32	1.17	0.94	1.15	0.98	1.20										16.13	5.72	27.66	10.71	17.28						20	
beta-centractin	IPI00153990.1	376	1	X	X	0	19.86	13	11	22.07	1.17	1.00	1.13	1.00	1.15	16.39	11	10	23.4	1.19	1.03	1.13	0.95	1.14	20.07	6.60	19.78	9.66	14.56	11.84	20.31	9.39	6.21	26.78	6	5
farnesyl pyrophosphate synthase isoform 2	IPI00120457.1	353	3	X	X	0	49.71	12	9	19.55	1.17	1.05	1.22	0.98	1.26	53.93	13	9	21.3	1.20	1.00	1.21	0.93	1.17	27.36	14.53	15.20	27.28	18.95	13.92	7.11	9.24	8.18	32.38	11	9
ganglioside GM2 activator precursor	IPI00119095.3	193	11	X		0	21.2	6	4	18.65	1.17	0.96	1.02	1.06	1.20										28.38	12.27	23.03	17.94	12.33						6	
myosin-9	IPI00788324.1	1960	15	X		0	715.99	114	99	37.76	1.17	1.00	1.20	1.00	1.17										19.96	12.52	20.46	11.56	21.00						172	
syntaxin-7	IPI00329953.1	261	10		X	0										63.62	11	8	24.1	1.17	0.99	1.15	1.04	1.14						23.22	6.49	27.22	8.28	20.09		12
LIM and SH3 domain protein 1	IPI00125091.1	263	11	X	X	0	97.88	23	17	48.29	1.16	0.94	1.18	0.97	1.19	126.8	19	13	39.9	1.19	1.07	1.28	1.02	1.19	13.41	8.06	18.41	11.62	21.16	24.20	12.23	31.12	12.71	21.67	31	35
catenin (cadherin associated protein), delta 1, isoform CRA_a	IPI00676489.1	932	2	X		0	102.45	27	26	21.03	1.16	1.02	1.19	1.05	1.17										24.81	10.75	21.47	13.45	11.03						28	
isoform 2 of dynactin subunit 1	IPI01008599.1	1243	6	X		0	119.5	48	38	25.34	1.16	1.03	1.15	0.97	1.10										21.25	14.32	17.58	17.17	15.56						21	
asparagine--tRNA ligase, cytoplasmic isoform 1	IPI00349070.1	559	18		X	0										67.19	20	18	23.8	1.16	1.06	1.19	0.85	1.04						19.82	13.28	34.73	5.74	27.40		12
plectin isoform 1a	IPI01008579.1	4543	15		X	0										2541	319	271	46.2	1.16	0.99	1.17	0.99	1.17						24.69	11.91	22.59	9.57	21.97		373
prelamin-A/C isoform A precursor	IPI00620256.3	665	3		X	0										1428	74	53	59.4	1.16	1.02	1.14	1.02	1.16						25.94	10.65	21.28	10.19	20.39		126
serine (or cysteine) proteinase inhibitor, clade B, member 9b	IPI00119079.6	377	13		X	0										156.8	12	10	22.6	1.16	1.02	1.24	1.01	1.22						23.42	8.15	23.00	10.23	28.76		13
asparagine synthetase [glutamine-hydrolyzing]	IPI00116966.8	561	6		X	0										29.04	17	16	21.6	1.16	0.94	1.23	0.99	1.22						22.05	13.36	24.83	5.36	17.77		10
proteasome subunit beta type-1 precursor	IPI00113845.1	240	17	X		0	41.45	6	5	22.5	1.15	0.99	1.15	1.03	1.26										9.94	9.07	12.57	13.79	3.35						6	
2-oxoglutarate dehydrogenase, mitochondrial isoform 3	IPI00626237.2	1023	11	X		0	70.04	23	20	17.01	1.15	1.08	1.09	0.98	1.14										15.51	9.59	16.04	9.74	11.57						22	
unnamed protein product = Coatomer subunit gamma-2	IPI00896657.1	870	6	X		0	214.65	19	17	17.24	1.15	1.02	1.17	1.00	1.09										23.98	11.56	16.00	4.98	17.74						13	
vesicle-trafficking protein SEC22b precursor	IPI00114368.3	215	3	X		0	114.09	22	12	41.4	1.15	0.98	1.17	1.00	1.11										31.68	17.89	22.71	9.18	12.41						8	
annexin A4	IPI00877291.1	319	6	X	X	0	399.7	22	20	50.47	1.15	0.87	1.15	0.92	1.24	436.8	26	19	47.3	1.30	1.01	1.29	0.97	1.42	22.83	9.16	20.22	12.81	30.59	34.33	12.58	17.34	15.13	33.81	37	42
brain-specific angiogenesis inhibitor 1-associated protein 2-like protein 1	IPI00321643.4	514	5	X	X	0	58.86	16	13	22.76	1.15	1.02	1.19	0.98	1.13	80.09	19	14	23.2	1.18	0.95	1.30	0.97	1.32	15.99	17.49	20.78	23.61	17.18	20.61	20.61	15.94	16.38	15.39	10	10
filamin-C	IPI00753917.2	2704	6	X	X	0	158.02	57	48	15.68	1.15	1.11	1.10	1.11	1.07	105.5	53	46	15.3	1.16	1.02	1.13	1.00	1.14	24.84	9.15	32.99	8.89	20.94	19.68	10.17	22.94	20.51	15.30	22	17
HAI-2 related small protein	IPI00110641.1	114	7	X	X	0	58.39	3	3	35.09	1.15	0.91	1.34	0.96	1.29	33.99	4	4	34.2	1.26	1.00	1.50	1.00	1.44	20.92	8.97	29.49	8.94	30.94	26.44	12.87	16.48	15.07	36.71	12	13
actin-related protein 3	IPI00115627.4	418	1	X	X	0	162.09	20	14	29.43	1.15	0.99	1.20	1.06	1.12	215.7	16	12	26.6	1.22	0.97	1.16	0.95	1.23	17.99	9.62	13.21	14.92	19.57	18.43	11.08	30.90	17.34	19.75	25	23
peroxisomal multifunctional enzyme type 2	IPI00408421.1	735	18	X		0	302.98	29	26	29.12	1.15	1.00	1.03	1.05	1.13										23.00	16.10	21.16	11.52	19.98						32	
endophilin-B2 isoform 2	IPI00153832.1	395	2		X	0										89.79	15	8	19.2	1.15	0.91	1.37	0.93	1.36						37.09	13.51	23.55	5.89	46.15		6
ribosome-binding protein 1	IPI00121149.1	1605	2		X	0										633.7	124	89	60.8	1.15	1.04	1.12	1.01	1.09						22.12	15.40	20.52	10.54	17.75		167
actin-related protein 2/3 complex subunit 2	IPI00661414.2	300	1		X	0										158.3	21	16	42.7	1.15	1.03	1.10	0.99	1.11						10.96	10.04	11.45	11.40	9.73		25
ubiquitin-associated protein 2	IPI00457533.2	1132	4		X	0										118.9	22	18	12.1	1.15	1.01	1.19	1.01	1.19						13.28	13.92	15.50	17.90	19.52		18
tight junction protein ZO-1 isoform 2	IPI00944062.1	1685	7		X	0										227.7	57	42	22.4	1.15	0.99	1.14	0.99	1.11						16.49	10.49	16.52	15.37	18.68		38
ras-related protein Rab-1B	IPI00133706.1	201	19	X		0	155.2	13	10	42.79	1.14	1.06	1.12	1.04	1.16										17.47	12.48	19.44	10.43	18.06						26	
protein S100-A13	IPI00124115.1	98	3	X	X	0	67.7	8	7	62.24	1.14	1.00	1.08	0.96	1.06	45.17	7	6	54.1	1.26	1.03	1.12	0.98	1.18	4.71	5.09	9.49	7.40	9.47	12.64	6.10	8.91	3.97	18.39	14	8
moesin	IPI00110588.4	577	X	X	X	0	264.82	55	49	49.39	1.14	1.00	1.13	1.02	1.16	253.1	62	51	47.1	1.20	1.01	1.22	1.03	1.26	16.94	9.55	18.78	11.09	19.95	20.59	12.14	22.96	8.58	23.38	101	84
alanine--tRNA ligase, cytoplasmic	IPI00321308.4	968	8	X		0	102.17	26	21	18.18	1.14	0.97	1.17	1.00	1.17										16.68	12.43	24.99	10.36	13.52						40	
E3 ubiquitin/ISG15 ligase TRIM25	IPI00649526.2	634	11	X		0	89.93	15	13	17.19	1.14	1.03	1.08	0.98	1.15										11.75	11.02	8.00	15.37	6.29						7	
signal recognition particle 72	IPI00316133.7	671	5	X		0	94.93	21	16	20.86	1.14	0.86	1.05	0.94	1.28										10.31	18.16	13.43	18.19	20.88						8	
insulin degrading enzyme	IPI00828796.1	1019	19	X		0	75.75	42	33	25.22	1.14	0.96	1.17	0.98	1.19										18.39	9.90	22.84	12.36	19.46						20	
integrin beta-1 precursor	IPI00132474.3	798	8	X		0	97.75	22	17	17.42	1.14	1.08	1.20	0.98	1.16										19.67	13.96	18.53	16.89	14.48						21	
dnaJ homolog subfamily A member 1	IPI00132208.1	397	4	X		0	202.96	14	13	25.94	1.14	0.96	1.12	0.99	1.13										14.67	10.94	24.46	8.48	13.92						26	
heterogeneous nuclear ribonucleoprotein H	IPI00133916.2	449	11	X		0	129.13	17	15	29.62	1.14	1.02	1.12	0.97	1.13										18.67	7.61	12.63	13.79	15.88						25	
myoferlin	IPI00989268.1	2048	19	X	X	0	460.54	89	68	30.18	1.14	1.04	1.10	1.04	1.09	541.5	88	70	30.4	1.18	1.03	1.13	1.01	1.12	21.19	13.02	16.49	12.74	16.42	18.61	10.51	18.75	9.95	14.09	108	91
optineurin	IPI00170101.1	584	2		X	0										243.3	35	25	38.4	1.14	1.01	1.12	0.99	1.17						26.27	17.07	21.01	15.72	31.22		25
lamina-associated polypeptide 2 isoform epsilon	IPI00828976.1	452	10		X	0										490.8	24	17	33.9	1.14	0.96	1.23	0.93	1.03						24.82	20.66	22.10	15.42	19.76		30
tumor protein D52	IPI00881225.1	206	3	X	X	0	119.77	16	12	39.81	1.13	0.94	1.11	0.93	1.15	124.8	12	10	39.3	1.14	1.03	1.06	0.95	1.19	29.75	11.05	21.50	11.55	32.26	30.98	12.78	27.28	6.13	38.86	14	15
unnamed protein product	IPI00224794.1	422	1	X	X	0	113.52	31	22	42.42	1.13	0.90	1.18	0.99	1.25	111.8	30	22	43.6	1.15	0.97	1.12	1.00	1.20	24.13	15.79	35.84	11.43	19.66	28.30	11.44	32.28	12.47	23.79	34	30
NADP-dependent malic enzyme isoform 1	IPI00881197.1	572	9	X		0	83.19	13	10	16.43	1.13	1.00	1.18	0.99	1.17										21.00	6.82	16.31	8.21	42.67						21	
alpha-actinin-1	IPI00380436.1	892	12	X	X	0	557.63	32	32	32.4	1.13	1.01	1.13	1.03	1.10	399.3	42	36	34.6	1.11	0.97	1.14	1.00	1.09	22.58	13.56	21.93	12.30	21.85	22.84	14.04	25.25	11.39	19.99	82	62
myosin light polypeptide 6	IPI00354819.5	151	10	X		0	174.99	12	7	47.68	1.13	1.00	1.15	1.06	1.09										22.61	9.75	18.50	9.24	18.15						28	
microtubule-associated protein	IPI00473748.5	1087		X		0	259.88	39	28	24.66	1.13	1.00	1.14	0.99	1.13										17.12	10.43	15.01	14.59	12.26						54	
mCG49732	IPI00849478.1	370	8	X		0	199.81	30	25	56.76	1.13	0.94	1.14	0.97	1.17										16.49	8.28	14.54	11.02	13.90						56	
PREDICTED: 40S ribosomal protein S2-like	IPI00458629.2	293	6		X	0										72.52	22	21	47.4	1.13	1.05	1.09	1.03	1.08						13.88	8.74	8.58	6.42	9.00		27
glycerol-3-phosphate dehydrogenase, mitochondrial	IPI00331182.4	745	2		X	0										68.7	22	17	21.5	1.13	0.99	1.14	0.99	1.16						15.74	11.26	12.51	6.96	22.93		16
serine/threonine-protein kinase PAK 2 precursor	IPI00229884.1	524	16		X	0										79.23	21	13	22.5	1.13	0.99	1.24	0.99	1.21						13.29	10.02	25.62	6.06	20.31		17
long-chain specific acyl-CoA dehydrogenase, mitochondrial	IPI00119114.2	430	1		X	0										85.51	23	20	37.9	1.13	0.98	1.06	0.94	1.07						15.53	8.11	8.61	10.38	12.25		27
adenine phosphoribosyltransferase	IPI00625950.2	180	8	X	X	0	86.12	7	7	36.11	1.12	0.95	1.17	1.02	1.15	44.97	8	8	37.8	1.24	0.93	1.32	0.94	1.47	12.96	14.87	9.42	10.20	11.23	14.36	4.50	4.30	3.24	11.02	14	5
IQ motif containing GTPase activating protein 1	IPI00467447.3	1657	7	X		0	487.12	78	58	29.51	1.12	0.98	1.13	1.00	1.10										21.79	11.27	22.98	15.48	16.63						87	
uncharacterized protein = nuclear mitotic apparatus protein 1	IPI00986204.1	1090	7	X		0	95.24	81	59	45.41	1.12	1.04	1.11	1.02	1.11										19.83	12.17	29.26	15.66	14.63						31	
glutamate dehydrogenase 1, mitochondrial precursor	IPI00114209.1	558	14	X		0	141.62	23	18	29.21	1.12	1.08	1.08	1.00	1.12										40.85	17.16	29.47	24.82	25.47						36	
transgelin-2	IPI00626106.3	199	1	X		0	258.86	18	13	64.32	1.12	0.96	1.08	0.96	1.19										19.25	10.78	24.57	10.45	22.26						30	
tropomyosin alpha-4 chain	IPI00421223.3	248	8	X		0	330.89	24	23	55.24	1.12	1.07	1.17	1.06	1.16										26.25	12.60	16.25	11.81	22.29						49	
catenin delta-1 isoform 4	IPI00660602.1	911	2	X	X	0	97.76	27	26	21.41	1.12	1.01	1.13	1.04	1.13	114.4	32	29	24	1.04	1.02	1.10	1.01	1.09	24.97	12.49	19.12	14.42	12.54	20.21	6.74	11.97	5.83	16.33	30	26
chloride intracellular channel protein 1	IPI00130344.3	241	17	X		0	261.84	9	8	29.46	1.12	0.93	1.06	0.99	1.10										15.14	7.36	27.92	10.72	18.13						23	
isoleucine--tRNA ligase, cytoplasmic	IPI00943445.1	1262	13	X		0	73.47	32	29	19.02	1.12	0.99	1.05	1.00	1.12										26.65	14.53	26.63	12.80	27.45						22	
TATA-binding protein-associated factor 2N	IPI00264652.3	557	11		X	0										181.3	25	22	31.8	1.12	1.02	1.13	1.03	1.12						9.66	13.31	22.25	14.01	16.15		20
N(G),N(G)-dimethylarginine dimethylaminohydrolase 1	IPI00109482.3	285	3	X		0	52.69	7	6	22.11	1.11	0.99	1.19	1.00	1.16										9.49	13.48	16.39	4.89	12.18						6	
sodium/potassium-transporting ATPase subunit alpha-1 precursor	IPI00311682.5	1023	3	X		0	141.96	33	26	23.85	1.11	1.03	1.13	1.01	1.11										29.60	12.14	25.56	13.31	18.18						49	
glutathione S-transferase Mu 1	IPI00230212.5	218	3	X		0	28.68	17	15	54.59	1.11	0.97	1.06	0.95	1.09										40.03	19.57	31.26	16.45	29.57						14	
destrin	IPI00127942.4	165	2		X	0										232.6	15	13	57	1.11	1.01	1.14	1.01	1.17						26.54	12.51	22.87	12.89	25.30		30
proteolipid protein 2	IPI00128982.1	152	13,X		X	0										27.15	4	2	9.87	1.11	0.97	1.15	0.94	1.06						14.08	1.32	14.91	1.85	4.02		5
Hnrpk protein	IPI00224575.1	464	13	X		0	242.9	25	21	34.48	1.10	1.04	1.04	0.96	1.06										17.20	11.94	16.62	11.96	14.01						51	
calmodulin	IPI00761696.2	149	2,17,7	X		0	39.16	7	7	34.9	1.10	1.02	1.09	1.02	1.02										19.53	12.69	15.45	10.75	21.06						26	
guanine nucleotide-binding protein G(I)/G(S)/G(T) subunit beta-1	IPI00120716.3	340	4	X	X	0	115.39	10	9	23.24	1.10	1.02	1.02	0.98	1.04	107.7	9	7	17.1	1.17	1.06	1.03	0.96	1.07	12.98	8.17	17.42	7.78	8.51	13.29	5.57	13.64	6.00	14.19	13	12
uncharacterized protein = 40S ribosomal protein S2-like	IPI00606379.2	293	12	X		0	121.99	26	22	49.83	1.10	1.01	1.08	1.01	1.06										11.18	8.80	10.54	9.51	10.26						30	
transketolase	IPI00137409.3	623	14	X		0	222.34	24	23	26.81	1.10	0.96	1.24	1.01	1.17										21.56	14.03	25.62	11.75	20.97						53	
transaldolase	IPI00124692.1	337	7	X	X	0	207.97	26	20	51.34	1.10	0.95	1.10	0.97	1.11	108.4	25	23	53.7	1.12	1.08	1.13	1.02	1.16	21.02	10.39	16.63	14.75	14.76	15.97	11.88	13.44	9.83	16.97	42	30
Serine/threonine-protein kinase PAK 1	IPI00132993.1	545	7	X	X	0	25.72	15	9	12.48	1.10	0.98	1.07	0.89	1.18	30.22	14	10	16.5	1.11	1.06	1.11	1.00	1.18	24.41	7.02	23.91	14.66	26.56	23.76	9.01	21.27	12.60	35.43	8	10
3'(2'),5'-bisphosphate nucleotidase 1	IPI00396778.1	308	1	X	X	0	92.47	12	9	27.92	0.97	0.93	1.07	0.99	1.07	87.27	11	7	24.4	1.11	0.98	1.22	1.02	1.22	15.69	6.22	10.78	14.68	20.92	9.91	10.48	12.92	7.18	15.55	13	13
protein Cald1	IPI00311344.4	768	6		X	0										187.2	54	47	46.4	1.10	1.00	1.17	1.03	1.12						19.08	17.64	17.72	14.55	22.12		41
charged multivesicular body protein 4b	IPI00171974.1	224	2	X	X	0	88.85	16	13	29.91	1.09	0.94	1.10	1.01	1.13	93.94	10	9	29.9	1.12	0.97	1.16	0.99	1.06	19.36	10.55	15.75	8.48	12.90	21.96	12.09	11.69	9.87	24.21	17	16

ras-related protein Rab-14	IPI00126042.3	215	2	X	X	0	117.36	11	8	41.4	1.09	0.99	1.04	0.99	1.15	78.36	11	8	35.8	1.12	0.97	1.18	1.04	1.02	14.90	10.96	26.04	12.40	17.33	30.55	13.84	10.88	4.44	28.49	17	11
sarcoplasmic/endoplasmic reticulum calcium ATPase 2 isoform a	IPI00468900.5	998	5	X	X	0	282.04	37	27	24.15	1.09	1.06	1.05	1.01	1.02	236.7	32	25	22.9	1.17	1.03	1.18	1.01	1.10	15.96	11.77	14.46	15.16	17.32	22.58	14.71	24.19	13.85	20.23	43	40
insulin-degrading enzyme	IPI00119784.1	1019	19		X	0										40.97	30	26	21.8	1.06	0.99	1.02	1.00	1.07						25.37	7.79	5.11	12.96	20.57		9
PDZ and LIM domain protein 5 isoform ENH1	IPI00828969.2	591	3	X	X	0	49.01	16	14	20.81	1.05	0.94	1.03	0.98	1.07	54.73	20	16	23.2	0.96	0.94	1.07	1.02	1.07	14.86	13.92	21.07	13.54	15.74	18.47	8.15	15.71	7.05	16.13	17	12
hypoxanthine-guanine phosphoribosyltransferase	IPI00284806.8	218	X	X	X	0	195.09	13	9	44.5	1.04	0.90	1.09	0.96	1.14	122.3	7	5	22.5	1.17	0.98	1.23	0.99	1.25	9.20	4.17	10.70	6.48	7.46	19.92	8.66	10.84	2.73	16.55	10	8
stathmin	IPI00551236.3	149	4	X	X	0	109.34	14	13	65.77	1.00	0.98	1.00	0.85	0.93	153.5	18	15	78.5	0.79	0.98	0.87	0.96	0.89	20.95	14.13	18.52	17.67	12.53	13.13	16.48	7.49	11.47	8.04	16	22
PREDICTED: CD63 antigen-like	IPI00986380.1	238	18	X	X	0	52.61	4	2	7.14	0.98	1.18	0.88	1.18	0.78	91.33	4	3	11.3	0.75	1.03	0.76	0.94	0.64	18.86	13.83	20.37	20.13	18.37	8.04	9.54	3.08	8.61	8.20	6	9
uncharacterized protein (Fragment) = TNF receptor-associated protein 1	IPI00987441.1	705	16	X	X	0	153.68	31	23	28.65	0.93	1.03	0.79	1.00	0.91	78.9	25	22	25.7	0.86	1.02	0.87	0.99	0.88	15.29	13.64	18.59	18.16	20.16	12.30	12.38	13.75	7.25	13.91	26	24
emerin	IPI00114401.1	259	X	X	X	0	59.8	6	4	18.15	0.91	1.01	1.00	1.03	0.96	60.37	7	7	23.6	0.86	0.98	0.90	0.98	0.81	11.05	10.38	20.30	18.45	11.14	12.85	6.30	5.40	12.39	14.23	11	12
fatty aldehyde dehydrogenase variant form	IPI00380755.1	507	11	X		0	9.03	13	12	19.92	0.90	0.95	0.85	1.02	0.87										24.62	5.57	16.84	3.68	19.58						4	
ATP synthase subunit beta, mitochondrial precursor	IPI00468481.2	529	10	X		0	500.04	29	25	43.86	0.90	1.02	0.90	1.01	0.86										12.78	11.07	15.09	13.28	11.90						50	
RAF proto-oncogene serine/threonine-protein kinase	IPI00118101.1	648	6	X		0	7.07	17	14	16.2	0.90	1.01	0.70	0.96	0.84										19.41	3.93	15.39	5.93	27.87						3	
branched-chain-amino-acid aminotransferase	IPI00886399.1	388	7		X	0										19.32	6	5	10.8	0.90	1.02	0.72	1.02	0.94						8.81	3.90	4.04	10.98	2.00		3
heterochromatin protein 1-binding protein 3 isoform 5	IPI00896020.1	541	4		X	0										34.22	21	20	30.7	0.90	0.92	0.86	0.99	0.87						9.22	4.29	14.43	5.64	10.15		8
isoform 2 of CUGBP Elav-like family member 1	IPI00229237.1	482	2	X		0	27.78	10	9	12.66	0.89	0.97	0.86	0.97	0.89										9.00	5.49	9.06	5.15	8.01						12	
clustered mitochondria protein homolog	IPI00347394.7	1353	11	X		0	65.65	29	25	16.41	0.89	1.05	0.88	1.05	0.88										25.86	5.76	14.13	8.98	11.89						11	
apoptosis-inducing factor 1, mitochondrial isoform 1 precursor	IPI00129577.1	612	X	X		0	74.2	25	15	25.33	0.89	0.96	0.85	0.97	0.88										8.74	7.77	11.03	12.33	12.37						12	
N-alpha-acetyltransferase 50	IPI00753284.3	168	16	X		0	39.28	14	9	45.83	0.89	0.97	0.85	0.95	0.84										15.27	9.59	26.46	13.32	26.72						14	
glutaminase kidney isoform, mitochondrial isoform 2	IPI00671957.2	603	1	X	X	0	205.67	17	16	23.88	0.88	1.07	0.82	1.05	0.75	269.9	18	15	20.7	0.90	1.01	0.87	1.01	0.89	18.75	10.59	13.38	4.39	13.45	11.88	9.09	14.59	14.84	17.03	17	14
glyoxalase domain-containing protein 4	IPI01007883.1	279	11	X	X	0	48.49	10	9	26.52	0.88	0.96	0.93	1.00	0.92	24.15	10	10	32.3	0.85	0.95	0.89	0.95	0.88	9.86	8.99	11.77	10.00	15.88	3.83	9.86	2.86	5.29	6.10	15	12
cyclin-dependent kinase 9	IPI00114953.1	372	2	X		0	114.46	13	11	24.73	0.88	1.06	0.80	0.98	0.81										4.16	15.27	30.24	38.70	14.57						4	
vesicle-fusing ATPase	IPI00656325.2	744	11	X		0	33.4	37	26	25.27	0.88	1.03	0.91	1.04	0.83										19.71	12.04	17.78	13.55	12.80						14	
cytoskeleton-associated protein 4	IPI00223047.2	575	10	X	X	0	187.75	40	32	48.17	0.88	1.04	0.84	1.03	0.87	124.8	26	21	33	1.43	1.01	1.31	0.98	1.48	15.69	18.01	14.17	11.33	16.15	45.12	13.46	22.73	26.30	44.34	38	18
protein RRP5 homolog	IPI00551454.3	1862	19	X		0	33.01	43	38	15.47	0.88	1.04	0.86	0.93	0.79										16.00	17.83	8.88	22.96	7.73						8	
mitochondrial import receptor subunit TOM40 homolog	IPI00869452.1	361	7	X		0	40.51	13	9	24.1	0.88	0.99	0.86	1.01	0.84										11.12	11.78	11.53	10.83	12.61						17	
heterogeneous nuclear ribonucleoprotein A0	IPI00109813.1	305	13		X	0										102.3	11	10	34.4	0.88	1.01	0.89	0.97	0.89						8.44	12.01	5.91	10.46	9.03		16
proline-, glutamic acid- and leucine-rich protein 1	IPI00321597.6	1123	11		X	0										73.79	15	12	10.4	0.88	1.11	0.81	1.21	0.78						30.93	20.38	7.02	30.05	9.56		9
putative pre-mRNA-splicing factor ATP-dependent RNA helicase DHX15 isoform 2	IPI00128818.2	795	5		X	0										41.75	34	27	27	0.88	1.09	0.87	1.02	0.90						12.12	17.18	17.09	6.79	10.59		16
nucleolar RNA helicase 2	IPI00652987.3	851	10	X	X	0	297.69	44	36	36.08	0.86	1.06	0.85	1.03	0.82	201.5	45	38	35.5	0.93	1.00	0.98	1.01	0.91	12.70	13.90	12.03	15.18	14.81	13.30	8.01	10.14	8.17	13.73	72	61
aspartate--tRNA ligase, cytoplasmic isoform 1	IPI00222457.2	501	1	X	X	0	182.57	26	21	32.93	0.86	0.96	0.90	0.97	0.87	164.8	25	20	35.5	0.88	1.00	0.90	0.99	0.90	17.78	10.83	13.85	4.02	10.32	20.55	11.05	18.71	13.37	12.31	28	29
eukaryotic peptide chain release factor subunit 1	IPI00312468.5	437	18	X	X	0	76.25	14	13	30.66	0.86	1.00	0.90	0.99	0.89	71.11	13	10	24.7	0.96	1.04	0.92	0.97	0.90	6.44	7.52	8.58	12.83	13.08	15.40	5.96	11.90	7.61	5.42	14	18
transcription intermediary factor 1-beta	IPI00312128.3	834	7		X	0										62.13	28	23	20.5	0.86	1.00	0.83	1.06	0.86						9.48	10.80	13.17	13.01	11.43		26
EH domain-containing protein 2	IPI00402968.1	543	7		X	0										66.45	24	19	29.3	0.86	1.05	0.86	1.04	0.88						13.78	11.02	13.09	9.22	17.21		18
protein jagunal homolog 1 isoform 1	IPI00762616.2	183	6		X	0										39	2	2	10.4	0.86	0.97	0.93	1.01	0.86						12.62	12.74	5.29	1.78	9.15		6
cytochrome b-c1 complex subunit 7	IPI00132347.1	111	13		X	0										41.27	4	4	27.9	0.86	1.04	0.92	1.02	0.89						16.34	11.72	16.97	5.68	19.30		5
mitochondrial import receptor subunit TOM70	IPI00988528.1	611	16	X	X	0	58.61	14	12	13.75	0.85	1.08	0.83	1.06	0.81	55.41	11	9	11.1	0.93	1.05	1.00	1.01	0.93	10.47	11.46	9.69	9.91	11.03	16.08	5.17	19.87	8.11	11.44	17	12
calponin-2	IPI00116649.1	305	10	X		0	48.96	15	11	28.52	0.85	0.93	0.92	0.92	0.87										4.11	4.39	7.63	13.50	10.30						7	
E3 ubiquitin-protein ligase HUWE1	IPI00655012.2	4377	X	X	X	0	123.25	80	62	11.19	0.85	1.03	0.79	1.00	0.82	201.9	68	60	11.2	0.93	1.05	0.85	1.00	0.94	15.33	15.73	8.63	10.51	14.88	33.17	12.75	22.05	20.42	17.92	14	16
galectin-1	IPI00229517.5	135	15		X	0										67.56	7	6	35.6	0.85	1.00	0.91	0.97	0.83						23.63	18.51	12.10	10.42	11.17		25
tropomyosin beta chain isoform 3	IPI00874728.1	284	4		X	0										192.9	23	20	50.4	0.85	1.03	0.90	1.01	0.85						20.68	8.79	28.06	11.64	18.79		36
pantetheinase precursor	IPI00943453.1	512	10	X	X	0	85.79	10	9	15.43	0.84	1.02	0.75	1.02	0.75	67.32	6	5	11.1	1.24	0.96	1.13	1.00	1.17	13.28	9.57	12.82	7.25	11.01	31.66	13.30	19.23	6.43	27.68	20	13
protein arginine N-methyltransferase 1	IPI00974841.1	318	7	X	X	0	69.04	6	6	15.09	0.84	0.96	0.87	1.00	0.85	23.77	7	7	16.7	0.90	0.96	0.99	1.04	0.90	7.52	7.05	15.10	5.33	11.00	3.57	2.39	12.32	11.99	11.35	10	7
vinculin	IPI00405227.3	1066	14	X	X	0	1058.4	88	56	46.15	0.84	0.97	0.85	0.96	0.87	905.8	82	58	47.2	0.80	0.99	0.85	1.00	0.83	11.32	9.38	12.55	12.23	11.37	12.61	9.04	12.51	6.98	11.57	125	120
ferritin heavy chain	IPI00230145.5	182	19	X	X	0	42.32	12	8	28.57	0.83	1.01	0.83	1.02	0.94	25.3	12	8	33.5	0.79	1.01	0.81	0.97	0.76	22.21	22.04	11.23	5.04	21.13	22.68	12.99	17.19	15.84	24.62	7	7
reticulon-4 isoform A	IPI00469392.2	1162	11	X	X	0	190.49	19	16	12.13	0.83	1.00	0.84	0.97	0.85	90.26	18	13	10.3	0.90	0.99	0.82	0.99	0.85	18.11	22.90	18.57	12.38	14.47	12.10	12.86	10.78	12.71	14.17	17	8
reticulon-4 isoform B1	IPI00470167.3	356	11	X		0	208.56	9	7	21.63	0.83	1.00	0.88	1.01	0.85										18.82	28.01	21.54	9.65	14.86						15	
NADH dehydrogenase [ubiquinone] 1 beta subcomplex subunit 5, mitochondrial precurso	IPI00132531.1	189	3		X	0										79.65	5	5	30.2	0.83	1.01	0.80	1.03	0.78						9.41	3.57	5.48	9.67	7.01		5
trifunctional purine biosynthetic protein adenosine-3	IPI00323644.5	1010	16	X	X	0	44.74	32	29	23.86	0.82	0.98	0.90	0.99	0.92	68.05	27	23	21.6	0.84	0.98	0.90	1.02	0.89	19.55	7.89	17.38	12.69	15.25	10.17	16.90	11.65	9.13	10.62	21	27
putative gag-pol protein	IPI00420814.3	1067	18		X	0										138.8	27	21	17.8	0.82	0.94	0.84	0.96	0.78						19.46	14.90	23.82	13.86	15.26		14
GMP synthase [glutamine-hydrolyzing]	IPI00351252.4	693	3		X	0										60.6	18	15	18.6	0.81	1.00	0.94	0.99	0.91						6.97	10.21	6.44	10.27	9.15		11
serpin H1 precursor	IPI00880252.1	417	7		X	0										98.05	18	17	38.6	0.81	1.00	0.84	0.96	0.84						8.91	10.84	12.00	11.97	10.35		25
proteasome activator complex subunit 1	IPI01026657.1	238	14	X	X	0	40.75	7	7	27.73	0.80	1.03	0.88	1.00	0.91	66.04	11	9	34.5	0.80	0.93	0.84	0.99	0.84	10.89	12.15	5.38	2.33	11.31	8.33	4.93	10.56	6.60	21.49	10	7
epoxide hydrolase 1	IPI00831534.1	441	1	X		0	97.97	22	16	28.57	0.80	1.01	0.76	0.98	0.86										15.84	11.38	15.51	7.96	22.80						27	
acidic leucine-rich nuclear phosphoprotein 32 family member A	IPI00314736.3	247	9		X	0										26.9	14	12	28.3	0.80	0.98	0.93	0.99	0.93						14.00	7.30	16.21	9.95	8.07		10
glutathione S-transferase omega-1	IPI00114285.1	240	19	X		0	223.35	18	16	50	0.79	0.91	0.83	0.98	0.85										22.08	13.16	18.07	12.52	20.67						44	
phosphate carrier protein, mitochondrial precursor	IPI00124771.1	357	10	X	X	0	66.93	17	13	28.01	0.79	1.04	0.81	1.05	0.76	33.84	17	14	26.9	0.94	1.02	0.93	1.01	0.88	11.87	6.86	10.95	9.33	11.20	7.84	7.44	8.48	8.05	9.38	13	11
peptidyl-prolyl cis-trans isomerase D	IPI00132966.3	370	3	X	X	0	105.24	16	14	30.27	0.79	0.93	0.84	0.93	0.84	74.68	17	14	29.2	0.85	1.02	0.88	1.04	0.91	13.10	4.65	15.21	7.48	13.97	10.71	8.85	6.64	7.47	9.45	19	20
catechol-O-methyltransferase	IPI00132076.1	265	16		X	0										153.9	6	4	11.3	0.79	0.94	0.83	1.00	0.80						11.71	6.41	8.94	13.39	11.66		8
PREDICTED: peroxiredoxin-6 isoform X1	IPI00754071.1	200	1	X	X	0	198.5	8	7	30	0.77	0.86	0.82	0.95	0.86	282.7	8	7	33.5	0.81	0.92	0.89	1.04	0.90	11.23	7.49	11.28	7.12	11.33	15.65	9.94	18.82	20.36	14.68	22	19
prostate apoptosis response 4 isoform P33	IPI00816941.1	289	10	X	X	0	79.26	8	7	23.88	0.78	1.11	0.84	1.07	0.84	91.72	8	7	24.2	0.75	1.10	0.76	1.18	0.81	18.61	19.47	17.20	32.06	18.81	28.43	15.51	9.37	18.70	11.88	11	10
aldose reductase	IPI00331729.3	316	6	X	X	0	88.15	22	17	37.03	0.75	0.88	0.81	0.96	0.81	43.37	18	17	38.3	0.80	0.96	0.87	1.02	0.88	14.26	17.20	15.62	9.71	12.99	11.71	9.47	12.64	7.81	7.04	27	22
DNA topoisomerase 2-beta	IPI00380494.1	1612	14	X	X	0	28.71	46	40	17.93	0.75	1.00	0.76	1.03	0.80	36.85	55	49	23.4	0.80	1.03	0.86	1.05	0.81	8.99	10.73	15.93	7.95	9.99	17.44	9.47	15.42	9.55	13.82	18	18
DNA topoisomerase II	IPI00122223.1	1528	11	X	X	0	164.75	72	63	29.25	0.74	1.04	0.77	1.03	0.77	154.6	70	57	28.2	0.84	1.01	0.87	1.04	0.82	14.25	9.89	18.54	9.91	17.42	15.16	8.77	13.94	11.21	13.96	64	48
Isoform B of Heat shock protein beta-1	IPI00468068.3	197	5		X	0										70.16	9	7	34	0.74	1.02	0.82	0.99	0.81						11.37	11.85	16.00	8.71	15.42		15
Fam3c protein	IPI00986569.1	311	6	X		0	26.64	11	10	27.01	0.77	0.90	0.73	0.95	0.85										21.14	4.88	22.96	2.61	17.05						2	
ceruloplasmin isoform b precursor	IPI00775908.1	1061	3		X	0										49.79	11	10	8.48	0.80	0.97	0.71	1.06	0.72						14.77	13.81	9.57	10.20	17.84		7
galactokinase	IPI00265025.5	392	11	X	X	0	88.18	9	8	18.11	0.72	0.85	0.78	0.94	0.78	75.9	12	10	21.7	0.84	0.97	0.85	1.02	0.93	3.84	6.83	6.55	5.78	12.11	16.48	17.88	12.66	8.03	12.68	7	8
transmembrane 4 L6 family member 1	IPI00133200.1	202	3	X	X	0	27.85	1	1	4.46	0.72	1.09	0.74	1.05	0.71	4.28	1	1	4.46	0.73	0.93	0.78	0.96	0.68	9.50	9.47	5.25	13.43	8.40	9.65	12.28	6.25	1.21	6.39	6	5
calponin-3	IPI00119111.2	330	3	X	X	0	61.37	21	14	30.91	0.87	0.90	0.83	0.95	0.83	135.4	18	12	27	0.62	0.96	0.63	0.94	0.66	18.60	10.15	12.72	12.41	21.60	18.48	7.24	23.53	12.93	18.59	13	18
cytosolic dioxin inducible aldehyde dehydrogenase-3	IPI00111222.1	453	11	X		0	19.4	11	11	20.53	0.62	0.95	0.65	1.05	0.75										9.36	5.60	7.08	3.39	13.49						4	
unnamed protein product = 3-oxoacid CoA transferase 1	IPI00858156.1	486	15		X	0										26.77	16	13	25.7	0.62	0.97	0.63	0.95	0.65						14.00	7.30	10.07	8.18	12.76		9
glutamine--fructose-6-phosphate aminotransferase [isomerizing] 1	IPI00755549.2	681	6		X	0										36.26	11	9	12.5	0.62	1.03	0.62	1.00	0.61						13.67	19.76	7.72	13.15	9.47		8
Aldh3a1 protein	IPI00890112.2	454	11		X	0										24.85	15	13	23.6	0.70	0.99	0.58	1.14	0.69						6.96	8.02	26.32	8.83	22.18		4
unnamed protein product = Prostaglandin G/H synthase 1 = Cox1	IPI00988446.1	488	2	X	X	0	68.42	8	7	12.7	0.65	1.07	0.56	1.01	0.64	48.73	12	11	18.4	0.96	1.09	0.83	1.01	0.85	9.74	5.13	7.58	11.44	15.88	15.73	0.15	10.87	3.69	5.55	8	3
prostaglandin G/H synthase 2 precursor = Cox2	IPI00308785.1	604	1	X		0	58.17	6	6	9.77	0.61	1.07	0.54	1.01	0.60										3.87	5.01	6.43	7.72	9.16						6	
annexin A6 isoform b	IPI00344395.1	667	11	X	X	0	101.82	39	37	41.83	0.74	0.95	0.77	0.97	0.77	71.77	42	39	46.6	0.50	1.02	0.54	0.97	0.52	27.39	9.26	24.41	13.39	18.27	16.06	11.33	22.52	9.17	22.25	34	34
ferritin light chain 1	IPI00608020.4	183	13,4,7	X	X	0	63.45	13	9	50.27	0.62	0.97	0.53	1.03	0.48	65.53	8	4	25.1	1.47	1.07	1.60	0.98	1.49	25.11	9.09	8.70	8.24	3.46	12.64	9.91	9.73	11.36	12.65	9	7
H1 histone family, member X	IPI00118590.1	188	6		X	0										132.8	12	9	34.6	0.48	0.92	0.61	0.88	0.46						8.92	11.04	20.96	10.63	6.35		8
extracellular superoxide dismutase [Cu-Zn] precursor	IPI00114319.1	251	5	X	X	0	17.07	8	4	11.55	0.57	0.86	0.45	0.82	0.64	30.34	3	2	8.37	0.75	0.85	0.60	0.94	0.75	19.68	7.01	3.13	4.95	14.80	23.39	55.19	22.83	12.78	13.78	3	3
